# Bioinorganic Chemistry Meets Microbiology: Copper(II) and Zinc(II) Complexes Doing the Cha-Cha with the C-t-CCL-28 Peptide, Dancing till the End of Microbes

**DOI:** 10.1021/acs.inorgchem.4c02500

**Published:** 2024-10-01

**Authors:** Klaudia Szarszoń, Natalia Baran, Paulina Śliwka, Magdalena Wiloch, Tomasz Janek, Joanna Wątły

**Affiliations:** † Faculty of Chemistry, University of Wrocław, F. Joliot-Curie 14, 50-383 Wrocław, Poland; ‡ Institute of Physical Chemistry, Polish Academy of Sciences, Kasprzaka 44/52, 01-224 Warsaw, Poland; § Department of Biotechnology and Food Microbiology, Wrocław University of Environmental and Life Sciences, Chełmońskiego 37, 51-630 Wrocław, Poland

## Abstract

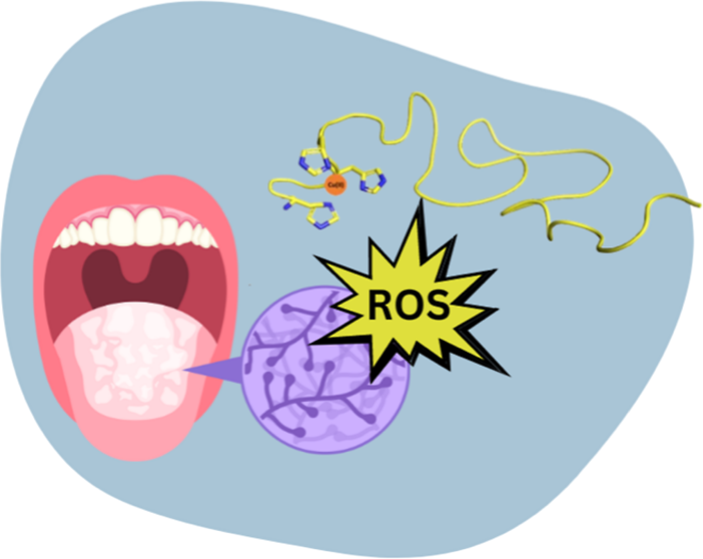

The necessity to move away from conventional antibiotic therapy has sparked interest in antimicrobial peptides (AMPs). One fascinating example is human CCL-28 chemokine produced by acinar epithelial cells in the salivary glands. It can also be released into the oral cavity with saliva, playing a crucial role in oral protection. The C-terminal domain of CCL-28 possesses antifungal and antibacterial properties, which are likely linked to membrane disruption and enzyme leakage. Studies suggest that AMPs can become more potent after they have bound Cu(II) or Zn(II). In many cases, these ions are essential for maximizing effectiveness by altering the peptides’ physicochemical properties, such as their local charge or structure. The examined peptide binds Cu(II) and Zn(II) ions very effectively, forming equimolar complexes. Metal ion binding affinity, coordination mode, and antimicrobial activity strongly depend on the pH of the environment. Coordination modes have been proposed based on the results of potentiometric titrations, spectroscopic studies (UV–visible, electron paramagnetic resonance and circular dichroism at different path lengths), and mass spectrometry. The antimicrobial properties of the Cu(II) and Zn(II) complexes with the C-terminal fragment of CCL-28 chemokine have been assessed against fungal and bacterial strains, demonstrating exceptional activity against *Candida albicans* at pH 5.4. Moreover, the complex with Zn(II) ions shows the same activity against the*Streptococcus mutans* bacterium as chloramphenicol, a commonly used antibiotic. Cyclic voltammetry proposed a probable antimicrobial mechanism of the studied Cu(II) complex through the formation of reactive oxygen species, which was also confirmed by tests with ascorbic acid in UV–vis and fluorescence spectroscopic studies.

## Abstract 2

Cu(II) and Zn(II) ions are clear boosters of the antifungal activity of the C-terminal peptide fragment of the chemokine CCL-28, an antimicrobial peptide produced by salivary glands. Metal binding significantly increases the antimicrobial potency of the peptide at slight acidic pH; Cu(II) coordination triggers ROS production, which is the most probable mechanism of antimicrobial action of the Cu(II)–C-t-CCL-28 complex.

## Introduction

Chemokines are predominantly small proteins with a low molecular weight (approximately ranging from 8 to 14 kDa). They are initially synthesized as pro-peptides, containing a signal peptide that undergoes cleavage to generate active or mature secreted proteins.^1,2^ They share conserved sequential and structural features, working alongside suitable receptors to guide the chemotactic activity of diverse immune cells across the body. In humans, over 45 chemokine members and 18 functional receptors have been discovered, which are engaged in various biological processes; they contribute to activities such as leukocyte degranulation, chemotaxis, angiogenesis, and hematopoiesis.^3,4^ Chemokines are typically classified into subfamilies based on the sequential arrangement of the first two out of four highly conserved cysteine residues: CXC, CC, C, and CX3C.^5^ Chemokines play crucial roles in both innate and adaptive immunity by controlling the migration and activation of leukocytes through a family of seven transmembrane G protein-coupled receptors.^1^ Most importantly, a number of chemokines have been acknowledged for their antimicrobial properties.^6^

Antimicrobial peptides (AMPs) are mostly cationic and amphipathic molecules, which are capable of directly eliminating pathogens by interacting with and/or penetrating through the negatively charged membranes of bacteria.^7−9^ As the majority of chemokines carry a positive charge at physiological pH, it is believed that a charge-based interaction is a significant aspect of their antimicrobial mechanism.^6^ AMPs can become even more potent when they coordinate with metal ions such as Cu(II) and Zn(II). In numerous instances, these ions are crucial for maximizing their effectiveness by modifying the peptides’ physicochemical properties, such as their local charge or structure.^10^ Moreover, the presence of transition metal ions such as copper(II) may lead to the formation of complexes capable of participating in the Fenton-like reaction, generating hydroxyl radicals. This process, involving the reduction of copper(II), results in the production of reactive oxygen species (ROS) and subsequent oxidative damage, including lipid peroxidation, protein oxidation, and DNA damage.^11−13^

**Figure 1:**
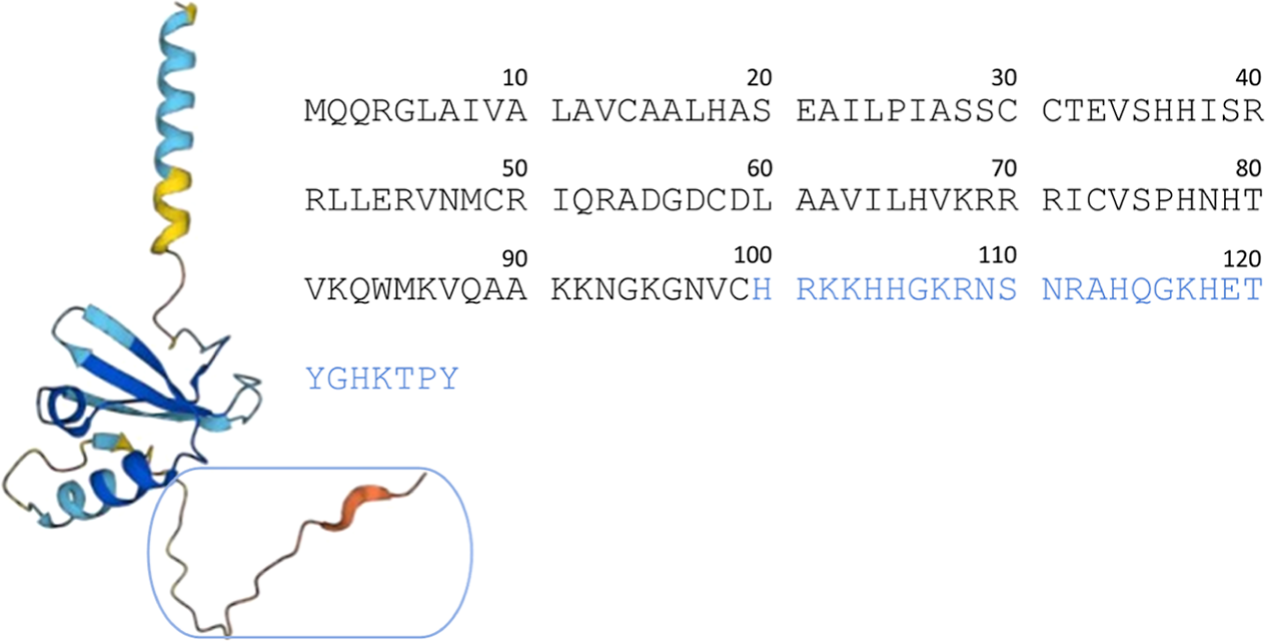
AlphaFold predicted structure and amino acid sequence of human CCL-28 chemokine (UniProtKB: Q9NRJ3). Analyzed C-terminal region of CCL-28 (HRKKHHGKRNSNRAHQGKHETYGHKTPY, C-t-CCL-28) is highlighted in blue.

CCL-28 (Figure 1), also known as mucosae-associated epithelial chemokine, is a chemokine found in various mucosal epithelial tissues.^14^ Transcripts of CCL-28 are found in various tissues, with the highest abundance observed in the trachea, colon, rectum, and exocrine glands such as mammary and salivary glands. CCL-28 produced by acinar epithelial cells in the salivary glands can also be released into the oral cavity along with saliva.^15^ CCL-28 is secreted in significant amounts in human saliva, ranging from 30 to 232 nM, as well as in milk, where concentrations typically fall between 13 and 34 nM.^15^ It not only plays a crucial role in several immune response pathways, particularly in the regulation of inflammation induced by cytokines,^15^ but also exhibits strong antimicrobial activity against a wide range of microbes, including *Candida albicans*, Gram-negative bacteria, and Gram-positive bacteria.^7^ Recently, studies showed that CCL-28 induces rapid damage to the membrane of *C. albicans* without needing active cellular processes.^16^ Interestingly, the antimicrobial properties of CCL-28 strongly rely on its C-terminal domain. It has been shown that removal of the C-terminal tail reduces the antifungal activity of CCL-28,^17^ and the peptide composed exclusively of CCL-28’s C-terminal domain maintains a considerable portion of the *in vitro* candidacidal activity observed in the full-length protein.^15^ Therefore, in this study, we decided to focus only on the C-terminal region (Figure 1—amino acid residues highlighted in blue).

**Figure 2:**
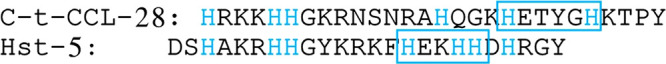
Comparison of the amino acid sequence of the C-t-CCL-28 and Hst-5 peptide. Zinc(II) binding motifs are marked with a blue frame.

The elongated C-terminal region of human CCL-28 (C-t-CCL-28) shares sequence similarity with histatin-5 (Hst-5), a candidacidal peptide rich in histidine residues that is secreted in human saliva [they share 53% sequence similarity and 32% sequence identity (Figure 2)].^15,18^ Both C-t-CCL-28 and Hst-5 possess binding sites highly tempting for Cu(II) and Zn(II) ions, including (i) histidine residues, (ii) the amide nitrogen from the peptide bond (in the case of Cu(II)),^19^ (iii) the typical zinc(II) binding motif (HEXXXH in CCL-28),^20^ and (iv) the HH motif, which is known to be linked to Cu(I/II)-peptide redox chemistry.^21^ In the case of CCL-28’s C-terminal fragment, the “histamine-like” binding site is also present, which involves two nitrogen atoms from the first histidine residue in the sequence (i) from the N-terminal amine group and (ii) from the imidazole ring.^22^

In this study, our attention is directed toward the C-t-CCL-28 peptide and its antimicrobial properties when forming complexes with Cu(II) and Zn(II). Our aim is to establish correlations among the coordination manner, thermodynamic characteristics, structural attributes, and the effectiveness of C-t-CCL-28 complexes against microbes while proposing potential mechanisms underlying their action.

## Experimental Section

### Materials

The C-terminal fragment of CCL-28 chemokine (HRKKHHGKRNSNRAHQGKHETYGHKTPY, C- and N-terminally free, named in this paper C-t-CCL-28) was purchased from KareBay Biochem (certified purity: 98%) and were used as received. The samples for electrospray ionization mass spectrometry (ESI-MS) were prepared in extra pure methanol (Sigma-Aldrich)-water mixture. Cu(II) and Zn(II) perchlorates were high-purity products [Cu(ClO_4_)_2_·6H_2_O from Sigma-Aldrich; Zn(ClO_4_)_2_·6H_2_O from POCH]. The concentrations of their stock solutions were determined by inductively coupled plasma mass spectrometry. The 0.1 M NaOH solution (EuroChem), which was free of carbonates, was standardized by using potentiometry with potassium hydrogen phthalate (Sigma-Aldrich). All of the samples were prepared with freshly double distilled water. The ionic strength (*I*) was adjusted to 0.1 M by the addition of NaClO_4_ (Sigma-Aldrich). Additionally, in the electron paramagnetic resonance (EPR) measurements, ethylene glycol (Chempure) was used. 50 mM phosphate buffer with pH 5.4 was prepared from a mixture of salts (Na_2_HPO_4_·2H_2_O and NaH_2_PO_4_·2H_2_O from POCH and EuroChem, respectively) in high-performance liquid chromatography-grade water. Additionally, Chelex 100 (Sigma-Aldrich) was added to minimize the influence of possible metal ions in the buffer on the experimental results. 50 mM phosphate buffer with pH 7.4 was prepared in an analogous manner. 20 mM stocks of ascorbate solutions were freshly prepared a few minutes prior to each experimental set by dissolving ascorbic acid (EuroChem) in phosphate buffer (pH 7.4). A 20 mM stock solution of coumarin-3-carboxylic acid (CCA) (from Sigma-Aldrich) was prepared using the phosphate buffer solution (50 mM, pH 7.4) at room temperature, and then the pH was lowered to pH 5.4. 4-Morpholineethanesulfonic acid (MES; Merck Millipore, Darmstadt, Germany) and 4-(2-hydroxyethyl)piperazine-1-ethanesulfonic acid (HEPES; Merck Millipore, Darmstadt, Germany) for biological studies were high-purity products. Commercial probes were used to stain cells for fluorescence microscopy: LIVE/DEAD Yeast viability Kit (L-7009, Invitrogen) and LIVE/DEAD BacLight Bacterial Viability Kit (L-7007, Invitrogen). 0.1 M KNO_3_ (Sigma-Aldrich, 99.99% trace metals basis) was used as a supporting electrolyte in voltammetric measurements. All samples were weighed using an analytical Sartorius R200D scale. All compounds used in the experiments were characterized by high purity (>95%).

### Mass Spectrometric Measurements

High-resolution mass spectra were acquired on an ESI-Q-TOF Maxis Impact (Bruker Daltonics) spectrometer, which measured Cu(II) and Zn(II) complexes (with both ligands) across both positive and negative ranges. The instrumental parameters were as follows: scan range *m*/*z* 50–3000; dry gas nitrogen; temperature 180 °C; capillary voltage 4000 V; ion energy 5 eV. The Cu(II) and Zn(II) complexes [(metal/ligand stoichiometry of 1:1) [ligand]_tot_ = 100 μM] were prepared in a 50:50 MeOH/H_2_O mixture at pH 6.5. The samples were infused at a flow rate of 3 μL/min. The instrument was calibrated externally with a Low Concentration Tuning Mix ESI-ToF (Agilent Technologies). The data were processed using Bruker Compass DataAnalysis 4.1 software.

### Potentiometric Measurements

The stability constants for proton and Cu(II) and Zn(II) complexes were determined from titration curves performed over the pH range of 2.0–12.0 at a temperature of 25 °C in a total volume of 2.7 cm^3^. The pH-metric titrations were performed in 0.004 M HClO_4_ with 0.1 M NaClO_4_ as the ionic strength using a Metrohm Titrando 809 titrator and a Mettler-Toledo InLab Micro combined glass electrode. The thermostabilized glass-cell was equipped with a magnetic stirring system, a microburette delivery tube, and an inlet–outlet tube for argon. The solutions were titrated with 0.1 M carbonate-free NaOH. The electrodes were calibrated daily for the hydrogen ion concentration by titrating HClO_4_ with NaOH in a total volume of 3.0 cm^3^. Calibration was conducted under the same experimental conditions as described above. The purities and exact concentrations of the ligand solutions were determined using the Gran method.^23^ The ligand concentration was 0.4 mM, and the Cu(II) and Zn(II) to ligand ratio was 0.9:1. The HYPERQUAD 2006 program was used for the stability constant calculations.^24^ Standard deviations were calculated using HYPERQUAD 2006 and were referenced only to random errors. The constants for the hydrolysis of Cu(II) and Zn(II) ions were obtained from the literature.^25,26^ The speciation and competition diagrams were computed with the HYSS program^27^ and visualized in the OriginPro 2016 program.

### Spectroscopic Studies

The absorption (UV–vis) spectra were recorded using a JASCO V-750 spectrophotometer, and the circular dichroism (CD) spectra were obtained with a JASCO J-1500 CD spectropolarimeter. Spectra were collected over the 220–800 nm range using a quartz cuvette with an optical path of 10 mm at a temperature of 25 °C in the pH range of 3.0–12.0. Direct CD measurements (Θ) were converted to mean residue molar ellipticity (Δε) using Jasco Spectra Manager. Far-UV CD spectra were recorded in the range of 180–250 nm in a 0.2 mm quartz cell at 25 °C for ligands and complexes at selected pH. The concentrations of solutions utilized for UV–vis and CD spectroscopic analyses were similar to those employed in the potentiometric experiments. The pH of the samples was regulated by adding suitable amounts of concentrated solutions of NaOH and HClO_4_, as needed. EPR spectra were obtained at liquid nitrogen temperature by using a Bruker ELEXSYS E500 CW-EPR spectrometer at a band frequency of 9.5 GHz. The tested ligands were prepared in aqueous solutions of HClO_4_ acid at *I* = 0.1 M (NaClO_4_), and ethylene glycol (30%) was added to the solutions as a cryoprotectant. The concentration of copper ions was 0.001 M and the metal/ligand ratio was 0.9:1. Measurements were conducted within the pH range of 3.0–11.0. The experimental EPR spectra were analyzed in order to ascertain the EPR parameters, which characterize the molecular and electron structures of the Cu(II) complexes, employing a simulation method. This method entailed finding the best fit between the theoretical and experimental spectra. The theoretical (simulated) EPR spectra were computed using the WinEPR SimFonia software, version 1.2 (Bruker), using the appropriately selected spin Hamiltonian EPR parameters for S = 1/2, including the diagonal components of the tensors: g (g∥ = gz, g⊥ = gx = gy) and A—interaction of an unpaired electron of copper(II) with a nuclear spin of copper, I(^63,65^Cu) = 3/2, (A∥ = Az, A⊥ = Ax = Ay). OrginPro 2016 was used to process and visualize the obtained spectra.

### Cyclic Voltammetry

Electrochemical experiments on glassy carbon disk (Ø 3 mm, Mineral) electrodes were performed using an Autolab PGSTAT204 potentiostat (Metrohm AG) controlled by the NOVA software (version 2.1.5). Cyclic voltammetry (CV) measurements were performed at a 20 mV/s scan rate, and at least 3 scans were recorded for each series. All experiments were performed in a three-electrode arrangement with a Ag/AgCl (3 M NaCl) reference electrode, a platinum rod as the counter electrode, and a glassy carbon disk electrode (GCE) as the working electrode. The reference electrode was separated from the working solution by a salt bridge filled with a 0.1 M KNO_3_ solution, the same pH as in the cell. The potential of the reference electrode was calibrated based on the ruthenium electrode process. The formal potential of hexaammineruthenium(III/II) chloride in 0.5 M KNO_3_ is 0.172 ± 0.002 V vs Ag/AgCl. Prior to each voltammetric measurement, the GCE was polished on a Buehler polishing cloth to a mirror-like surface, using aqueous slurries of 0.05 μm alumina powder followed by 1 min water ultrasonication to remove the remaining powder. All electrochemical measurements were carried out in 0.1 M KNO_3_ solution at pH 5.4 and 7.4. The pH was adjusted by a pH-metric titration with small volumes of concentrated NaOH solution. The concentration of metal-free C-t-CCL-28 in complexes was 0.3 mM. The copper(II)-to-ligand ratio was 0.9:1 in all cases [a small Cu(II) deficiency helps to avoid interference from uncomplexed Cu(II) cations]. The pH was closely monitored before and after each voltammetric measurement. The solution was saturated with argon during the experiments at 25 °C.

### HO^•^ Scavenging Monitoring

CCA was employed as a probe to detect hydroxyl radicals (HO^•^). This detection occurs because HO^•^ reacts with CCA to produce 7-hydroxycoumarin-3-carboxylic acid (7-OH–CCA), which exhibits fluorescence at 452 nm when excited at 395 nm. The fluorescence intensity is directly proportional to the quantity of 7-OH–CCA molecules formed, which correlates with the amount of HO^•^ radicals generated.^28^ The samples were prepared in 50 mM phosphate buffer at pH 5.4 (HEPES buffer is not preferred because HO^•^ may react with it). The final concentrations of the reagents in the cuvette were as follows: 60 μM (peptide), 50 μM (Cu(II)), 200 μM (CCA), and 200 μM (Asc). Ascorbate was added last, and the measurement started immediately. Fluorescence experiments were performed on an RF-6000 spectrofluorometer (Shimadzu). The fluorescence was measured every 30 s for 1 h (λ_exc_ = 395 nm, λ_em_ = 452 nm).

### Ascorbate Consumption Experiments

UV–vis spectra were recorded on a JASCO V-750 spectrophotometer at 25 °C. The intensity of the Asc absorption band at λ_max_ = 265 nm was monitored as a function of the time in 50 mM phosphate buffer solution at pH 5.4. The final concentrations of the reagents in the cuvette were as follows: 10 μM (peptide), 8 μM (Cu(II)), and 100 μM (Asc). The absorbance was measured every 10 s for 40 min after the addition of the peptide/Cu(II)/complex to Asc solution in buffer (control measurement was measured every 10 s for 10 min) using a quartz cuvette with an optical path of 10 mm. Each experiment was repeated three times to ensure the accuracy of the results.

### *In Vitro* Antimicrobial Activity of Peptides and Peptide–Metal Ion Systems

The peptide and its complexes were evaluated for their antimicrobial properties against human pathogenic strains. Four reference strains from American Type Culture Collection (ATCC), namely, *Escherichia coli* 10536, *Pseudomonas aeruginosa* 15442, *Enterococcus faecalis* 29212, and *Staphylococcus aureus* 25923, two from Polish Collection of Microorganisms (PCM), namely, *Streptococcus mutans* 2502 and *Streptococcus sanguinis* 2335, and *C. albicans* SC5314 were used for antimicrobial activity assay.^29^*E. coli* ATCC 25922, *P. aeruginosa* ATCC 15422, *E. faecalis* ATCC 29212, and *S. aureus* ATCC 25923 were grown at 37 °C in Mueller–Hinton broth (MHB) (Merck Millipore, Darmstadt, Germany). *S. mutans* PCM 2502 and *S. sanguinis* PCM 2335 were cultured in Brain Heart Infusion (BHI) broth (Merck Millipore, Darmstadt, Germany) and incubated overnight anaerobically (85% N_2_, 10% H_2_, and 5% CO_2_) at 37 °C. *C. albicans* SC5314 was grown aerobically at 37 °C on Yeast Peptone Dextrose (YPD) broth (A&A Biotechnology, Gdańsk, Poland).

### Bacterial Susceptibility Assay

The peptide/complexes minimal inhibitory concentrations (MICs) were determined using the serial broth microdilution method.^30^ Briefly, 2-fold serial dilutions of each peptide/metal(II) complex (molar ratio, 1:1) in MHB, BHI, and YPD broth buffered with 10 mM MES buffer, pH 5.4 (Merck Millipore, Darmstadt, Germany) or 10 mM HEPES buffer, pH 7.4 (Merck Millipore, Darmstadt, Germany), at a volume of 100 μL were prepared in 96-well flat-bottomed microtiter plates (Sarstedt, Nümbrecht, Germany). The final concentration of each peptide/complex was ranged from 7.8 to 500 μg/mL. The microtiter plate wells were inoculated with 1 μL per well of a 24 h culture of microorganisms at a final cell density of 5 × 10^7^ CFU/mL. Negative and growth control wells did not contain the tested compounds. Copper and zinc ions (10 μg/mL) served as negative controls, and in this instance, no antimicrobial activity was detected. Bacteria and *C. albicans* incubated with metal ions were used as additional controls. The antibacterial activity of a common standard antibiotic chloramphenicol (Merck Millipore, Darmstadt, Germany) and antifungal fluconazole (Thermo Fisher Scientific, Cleveland, OH, USA) was also recorded using the same procedure as above at concentrations ranging from 1 to 500 μg/mL. The microplates were incubated for 24 h at 37 °C for *E. coli* ATCC 25922, *P. aeruginosa* ATCC 15422, *E. faecalis* ATCC 29212, *S. aureus* ATCC 25923, and *C. albicans* SC5314. Two oral bacteria strains, *S. mutans* PCM 2502 and *S. sanguinis* PCM 2335, were incubated at 37 °C anaerobically (85% N_2_, 10% H_2_, and 5% CO_2_) and OD600 was measured after 72 h using a microplate reader (Spark, Tecan Trading AG., Switzerland). The MIC end point was determined as the lowest concentration resulting in complete (100%) growth inhibition. All assays were conducted in triplicate.

### Visualization of Cell Death

*S. mutans* PCM 2502 and *S. sanguinis* PCM 2335 harvested in the log phase were incubated in BHI medium, and *C. albicans* SC5314 harvested in the log phase was incubated in YPD medium with peptide/complexes. Microorganisms incubated without peptides or complexes were used as controls. After 24 h at 37 °C, microorganisms were washed with sterile MES (pH 5.4) buffer. Then, the bacterial cells were stained for 30 min in the dark at 37 °C with the LIVE/DEAD BacLight Viability Kit (L-7007, Invitrogen) prepared in MES buffer (pH 5.4), and the MES-containing LIVE/DEAD Yeast Viability Kit (L-7009, Invitrogen) was used for *Candida* cells. Cells were visualized using fluorescence microscopy (Axio Scope A1, Zeiss, Jena, Germany). Images were acquired and analyzed using Carl Zeiss ZEN 2.3 lite software for quantification of live and dead cells. Experiments were repeated three times.

## Results and Discussion

### Deprotonation Constants

Based on a series of potentiometric titrations, 15 deprotonation constants were established for the CCL-28 fragment (HRKKHHGKRNSNRAHQGKHETYGHKTPY, C-t-CCL-28) (Table 1). The determined values are in agreement with those reported in poly-His systems.^31−37^

**Table 1:** Deprotonation Constants (p*K*_a_) for C-t-CCL-28 Peptide and Stability Constants (log β) for Its Complexes with Cu(II) and Zn(II) Ions in Aqueous Solution of 4 mM HClO_4_ with *I* = 0.1 M NaClO_4_ at 25 °C^a^

C-t-CCL-28				Cu(II)–C-t-CCL-28			Zn(II)–C-t-CCL-28		
species	log β_jk_^b^	p*K*_a_^c^	residue	species	log β_jk_^d^	p*K*_a_^e^	species	log β_jk_^d^	p*K*_a_^e^
[H_16_L]^12+^	125.51(1)	2.46	COOH	[CuH_12_L]^10+^	115.32(1)		[ZnH_10_L]^8+^	101.43(2)	
[H_15_L]^11+^	123.05(1)	3.75	Glu	[CuH_11_L]^9+^	110.84(2)	4.48	[ZnH_8_L]^6+^	88.93(2)	
[H_14_L]^10+^	119.3(1)	4.89	His	[CuH_10_L]^8+^	106.15(2)	4.69	[ZnH_7_L]^5+^	81.12(4)	7.81
[H_13_L]^9+^	114.41(1)	5.59	His	[CuH_9_L]^7+^	100.31(2)	5.84	[ZnH_5_L]^3+^	63.71(4)	
[H_12_L]^8+^	108.82(1)	5.94	His	[CuH_8_L]^6+^	93.96(2)	6.35	[ZnH_3_L]^+^	44.2(5)	
[H_11_L]^7+^	102.88(1)	6.33	His	[CuH_6_L]^4+^	77.47(2)		[ZnH_2_L]	33.98(4)	10.22
[H_10_L]^6+^	96.55(1)	6.67	His	[CuH_4_L]^2+^	59.9(2)		[ZnL]^2-^	12.59(4)	
[H_9_L]^5+^	89.88(1)	7.21	His	[CuH_2_L]	40.47(3)				
[H_8_L]^4+^	82.67(1)	9.12	H_3_N^+^	[CuL]^2-^	19.84(2)				
[H_7_L]^3+^	73.55(1)	9.61	Tyr	[CuH_-2_L]^4-^	–2.21(4)				
[H_6_L]^2+^	63.94(1)	9.90	Tyr	[CuH_-3_L]^5-^	–13.38(4)	11.17			
[H_5_L]^+^	54.04(1)	10.28	Lys						
[H_4_L]	43.76(1)	10.49	Lys						
[H_3_L]^-^	33.27(1)	10.90	Lys						
[H_2_L]^2-^	22.37(1)		Lys						

a *C*_L_ = 0.4 mM; molar ratio M/L—0.9:1. The standard deviations are reported in parentheses as uncertainties on the last significant figure.

b Constants are presented as cumulative log β_jk_ values. β(H_j_L_k_) = [H_j_L_k_]/([H]^j^[L]^k^), in which [L] is the concentration of the fully deprotonated peptide.

c p*K*_a_ values of the peptides were derived from cumulative constants: p*K*_a_ = log β(H_j_L_k_) – log β(H_j–1_L_k_).

d Cu(II) and Zn(II) stability constants are presented as cumulative log β_ijk_ values. L stands for a fully deprotonated peptide ligand that binds Cu(II) and Zn(II) ions: β(M_i_H_j_L_k_) = [M_i_H_j_L_k_]/([M]^i^[H]^j^[L]^k^), where [L] is the concentration of the fully deprotonated peptide.

e p*K*_a_ = logβ (M_i_H_j_ + 1L_k_) – logβ(M_i_H_j_L_k_).

### Metal–C-t-CCL-28 Complexes

To explore the exact stoichiometry, structural characteristics, and thermodynamic properties of metal–C-t-CCL-28 complexes, a range of experimental techniques were applied. These methods included ESI-MS, a series of potentiometric titrations, and UV–vis, CD, and EPR spectroscopies.

The mass spectrometry measurements provided verification that the examined peptides have the capacity to form complexes with Cu(II) and Zn(II) in a 1:1 ratio (metal/ligand). No bis- or polynuclear or dimeric complexes were observed under experimental conditions, as confirmed by both potentiometry (Table 1) and ESI-MS results (Figures S1–S3). The most intense signals (*m*/*z*) for each system were identified and matched with those of the corresponding species. The signals and isotopic distributions in both the experimental and simulated spectra for the selected signals are consistent, confirming the accurate interpretation (Figures S1 and S2). Additional signals present in the displayed spectra primarily correspond to chloride and sodium adducts of both ligands and complex species as well as residual impurities within the measuring instrument.

### Cu(II)–C-t-CCL-28 Complexes

**Figure 3:**
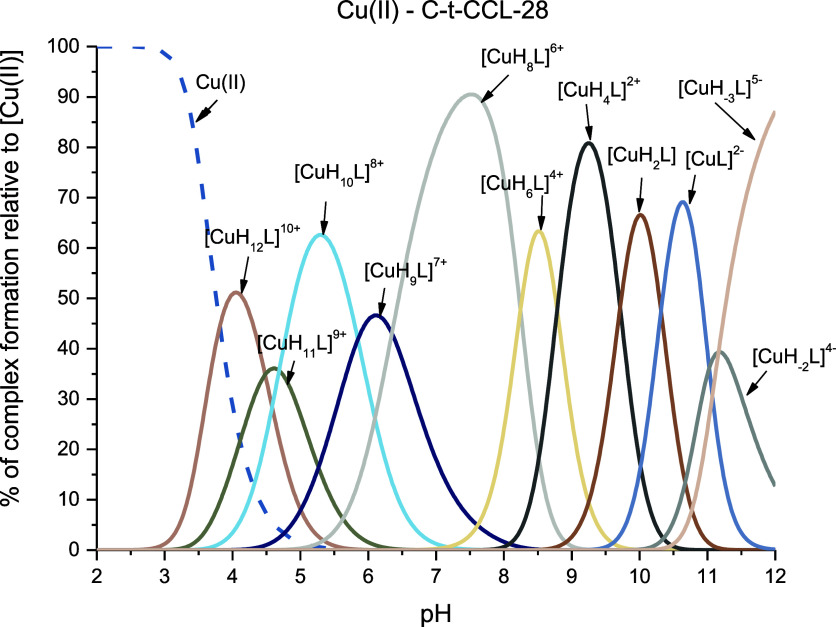
Representative distribution diagram for the Cu(II)–C-t-CCL-28 system in aqueous solution of 4 mM HClO_4_ with *I* = 0.1 M NaClO_4_ dependent on pH values. *C*_L_ = 0.4 mM; molar ratio M/L—0.9:1.

The stability constants of Cu(II) complexes with the C-t-CCL-28 peptide were determined by analyzing the titration curves recorded over a pH range of 2.0 to 12.0, as illustrated in Figure 3. The findings are presented in both Tables 1 and S1. Potentiometric measurements unveiled the existence of 11 equimolar complex species within the Cu(II)–C-t-CCL-28 system.

**Figure 4:**
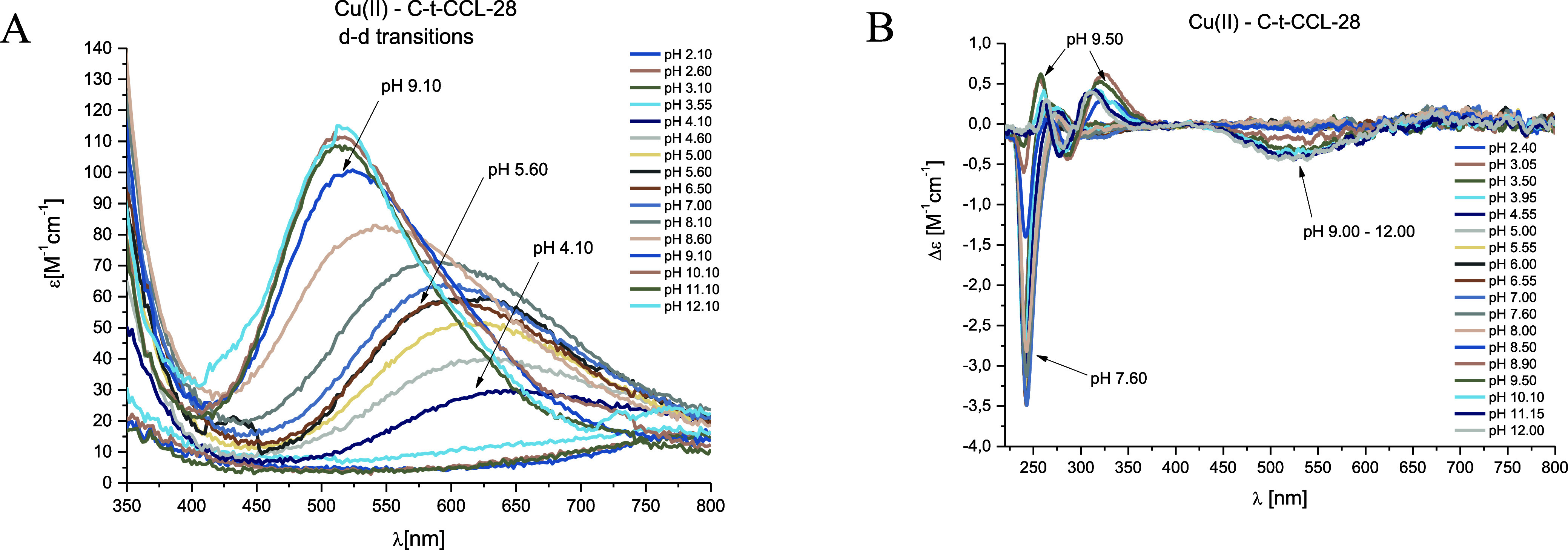
pH-dependent spectra: (A) UV–vis and (B) CD for the Cu(II)–C-t-CCL-28 system in aqueous solution of 4 mM HClO_4_ with *I* = 0.1 M NaClO_4_. Optical path length of 1 cm. *C*_L_ = 0.4 mM; molar ratio M/L—0.9:1.

The first complex observed for C-t-CCL-28 is [CuH_12_L]^10+^, with a maximum at pH 4.0 and most likely involves two histidine residues in copper(II) binding. 2 N coordination is confirmed by the d–d band in UV–vis spectra at 644 nm (Figure 4A) and EPR parameters (A∥ = 173 G, g∥ = 2.26) (Figure S4). The loss of the next proton leads to the [CuH_11_L]^9+^ complex species, with a maximum concentration at pH 4.7. The lowered histidine p*K*_a_ value in the complex in relation to the free ligand [p*K*_a_ 5.94 (ligand) → p*K*_a_ 4.48 (complex)] strongly suggests that another histidine residue is involved in the Cu(II) ion coordination. However, in the UV–vis and CD spectra, no significant changes are observed (Figure 4). This indicates that there are several different complex species in equilibrium, in which a maximum of two imidazole nitrogens are engaged in metal binding. This phenomenon is commonly referred to as polymorphic binding states.^38,39^ The next complex species, [CuH_10_L]^8+^, dominates in solution at pH 5.3. At this pH, a hypsochromic effect is observed in the UV–vis spectra, from 628 to 598 nm, and EPR parameters (A∥ = 180 G, g∥ = 2.25) suggest a 3N coordination mode with a {3N_im_} donor set. This binding mode remains unchanged in the next two complex species: [CuH_9_L]^7+^ and [CuH_8_L]^6+^, which are other forms with polymorphic binding states, but compared to the [CuH_11_L]^9+^ complex species, they have a maximum of three histidyl residues in the coordination sphere. Lowering the p*K*_a_ value of histidine residue (p*K*_a_ 6.67 → p*K*_a_ 5.84 for [CuH_9_L]^7+^ and p*K*_a_ 7.21 → p*K*_a_ 6.35 for [CuH_8_L]^6+^) and no noteworthy changes in spectroscopic data (Table S2) confirm this statement. The next complex species, [CuH_6_L]^4+^, dominates at pH 8.55 and is probably associated with deprotonation of the nonbinding N-terminal amine group (p*K*_a_ 9.12) and nonbinding Tyr residue (p*K*_a_ 9.61).

Above pH 8.5, an amide nitrogen starts to take part in the coordination, which is supported by the CD spectra with characteristic bands with a negative Cotton effect at 524 nm and a positive Cotton effect at 703 nm. In UV–vis spectra, a hypsochromic shift was observed from 542 to 522 nm, and EPR parameters at this pH were as follows: A∥ = 200 G and g∥ = 2.19. These parameters indicate a 4N coordination mode in case of the [CuH_4_L]^2+^ complex species with a {3N_im_, 1N_am_} donor set. Additional deprotonation probably comes from a nonbonding Tyr residue with p*K*_a_ 9.90. The loss of two next protons leads to the [CuH_2_L] complex species, with a maximum concentration at pH 10.05, which is probably related to the deprotonation of two amide groups leading to the formation of typical square planar complex with a {1N_im_, 3N_am_} donor set. The replacement of two imidazole residues with amide nitrogens from the peptide bond is supported by a hypsochromic shift in the UV–vis spectrum from 522 to 516 nm and broadening of the signal that corresponds to the negative Cotton effect at 238 nm. In the CD spectrum (Figure 4B), bands typical for square-planar complexes are observed. Above pH 10, the coordination mode remained the same. Formation of the next complex species: [CuL]^2–^, [CuH_–2_L]^4–^, and [CuH_–3_L]^5–^ is most probably related to the deprotonation of four nonbonding Lys residues (three with p*K*_a_ 10.28, p*K*_a_ 10.49, and p*K*_a_ 10.90, respectively, and one Lys residue, which was not detected during the ligand titration). The obtained relation of g tensor components, gz (corresponding to g||) ≫ gx = gy (corresponding to g⊥) > 2.0023 in the EPR spectra (Figure S4) proves axial symmetry of Cu(II) coordination spheres in all above-described copper complex species.^40^

**Figure 5:**
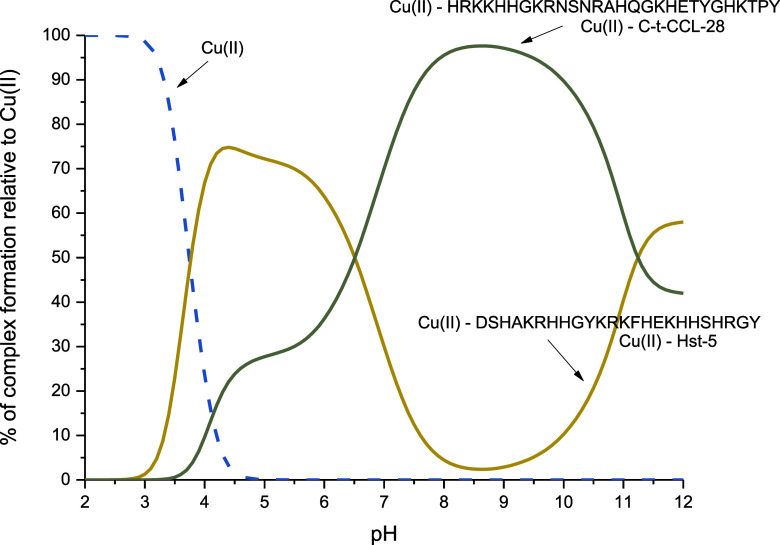
Competition plot for Cu(II) complexes with C-t-CCL-28 and Hst-5 based on potentiometric data (Table 1 and ref (36)), describing complex formation at different pH values in a hypothetical situation, in which equimolar amounts of the all reagents are mixed. Conditions: *T* = 25 °C, [Cu(II)] = [C-t-CCL-28] = [Hst-5] = 0.001 M.

We compare the stability of copper complexes of the Hst-5 peptide to the peptide studied in this work on a competition plot, based on the complexes’ stability constants, which shows a hypothetical situation in which equimolar amounts of the three reagents are mixed (Figure 5). The comparison shows that Hst-5 has a higher affinity toward Cu(II) from pH 3.0 to almost pH 7.0. Above pH 7.0, higher affinity of Cu(II) toward C-t-CCL-28 is observed. There are at least two reasons for that: (i) below pH 6.5, Hst-5 binds Cu(II) through the ATCUN motif, which forms highly stable complexes;^41^ and (ii) above pH 6.5, C-t-CCL-28 binds Cu(II) through polymorphic binding sites, which involves three imidazoles in the coordination mode. A situation in which polymorphic states form more stable complexes than the ATCUN binding mode was observed before in similar systems.^39,42^ Around pH 9.0, the coordination of Cu(II) to C-t-CCL-28 changes, involving a nitrogen atom from the amide group. At the same time, it is associated with a disturbance in the occurrence of polymorphic binding states, resulting in a decrease in the affinity of Cu(II) ions for C-t-CCL-28 in favor of Hst-5.

### Zn(II)–C-t-CCL-28 Complexes

**Figure 6:**
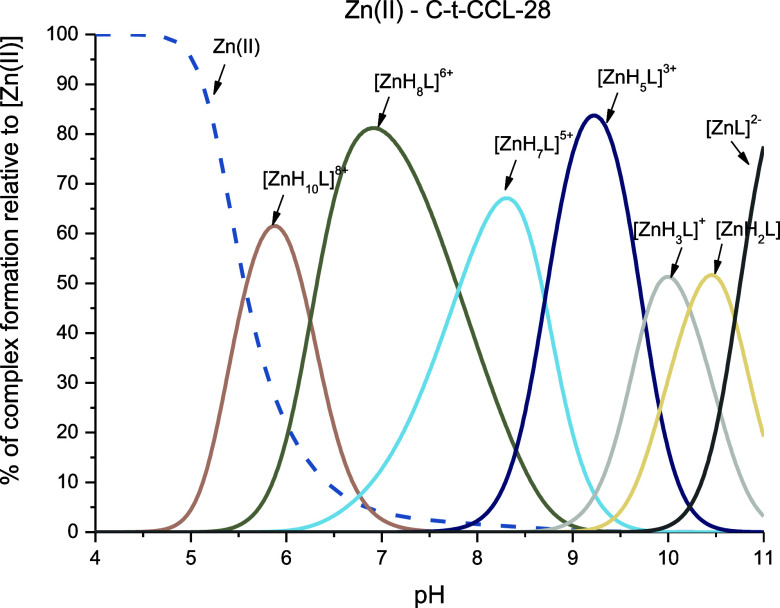
Representative distribution diagram for the Zn(II)–C-t-CCL-28 system in aqueous solution of 4 mM HClO_4_ with *I* = 0.1 M NaClO_4_ dependent on pH values. *C*_L_ = 0.4 mM; molar ratio M/L—0.9:1.

The best fit for the titration curve of the Zn(II)–C-t-CCL-28 system was achieved by considering the formation of seven complex species, with [ZnH_10_L]^8+^ occurring at the lowest pH. The findings from the potentiometric titrations are outlined in Table 1 and Figure 6.

In [ZnH_10_L]^8+^, all of the acidic groups and four imidazoles are already deprotonated. In this case, it is not possible to distinguish whether Zn(II) is bound to imidazole nitrogens from four His or to a set of two different histidyl residues, forming the so-called polymorphic binding states, as previously observed in the case of Cu(II) complexes. It is quite likely that in one of the two complexes present in solution, Zn(II) is bound to two His imidazoles and one Asp carboxylic side chain from the typical Zn(II)-binding HEXXXH motif; the other complex present in equilibrium may involve two imidazoles from other His residues in the peptide sequence. The loss of next two protons leads to the [ZnH_8_L]^6+^ species, which is related to deprotonation of two nonbonding His residues (p*K*_a_ 6.67 and p*K*_a_ 7.21, respectively). In the next complex, [ZnH_7_L]^5+^, with a maximum concentration at pH 8.3, most likely, the N-terminal amino group participates in binding, evidenced by a decrease in the p*K*_a_ of this group in the complex compared to the free ligand (p*K*_a_ 9.12 → p*K*_a_ 7.81). Most probably, in the studied system, also at higher pH, the presence of two different donor sets is observed: (i) one with at least one imidazole and the N-terminal amino group and (ii) one in which two imidazoles and the carboxylic acid from the side chain of Glu from the typical HEXXXH Zn(II) binding sites. Above pH 8, the [ZnH_5_L]^3+^, [ZnH_3_L]^+^, [ZnH_2_L], and [ZnL]^2–^ complexes dominate in solution, where the coordination mode remains the same. Their presence results from deprotonation of two Tyr residues with p*K*_a_ 9.61 and p*K*_a_ 9.90 and four nonbonding Lys residues.

According to the competition plot (describing the complex formation at different pH values in a hypothetical situation where equimolar amounts of all reagents are mixed) for the Cu(II) and Zn(II) complexes with the C-t-CCL-28 peptide (Figure S5), a huge difference in the binding affinity of these metal ions was observed, with Cu(II) ions being clearly more preferred in this system.

**Figure 7:**
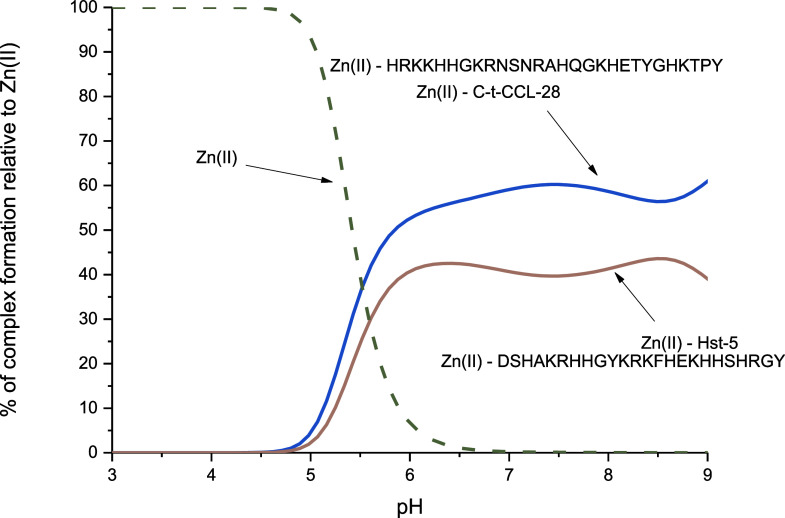
Competition plot for Zn(II) complexes with C-t-CCL-28 and Hst-5 based on potentiometric data (Table 1 and ref (36)), describing complex formation at different pH values in a hypothetical situation, in which equimolar amounts of all reagents are mixed. Conditions: *T* = 25 °C, [Cu(II)] = [C-t-CCL-28] = [Hst-5] = 0.001 M.

Upon comparison of the binding efficiency of Zn(II) ions by the salivary peptides Hst-5 and C-t-CCL-28, the competition plot (Figure 7) indicates comparable effectiveness in metal ion binding at pH values below 8.5. This phenomenon likely arises due to the presence of the HEXXH motif in Hst-5 and the HEXXXH motif in C-t-CCL-28, both of which are recognized as canonical zinc(II) ion-binding domains.^20^

### Impact of Metal Ion Coordination on the C-t-CCL-28 Secondary Structure

**Figure 8:**
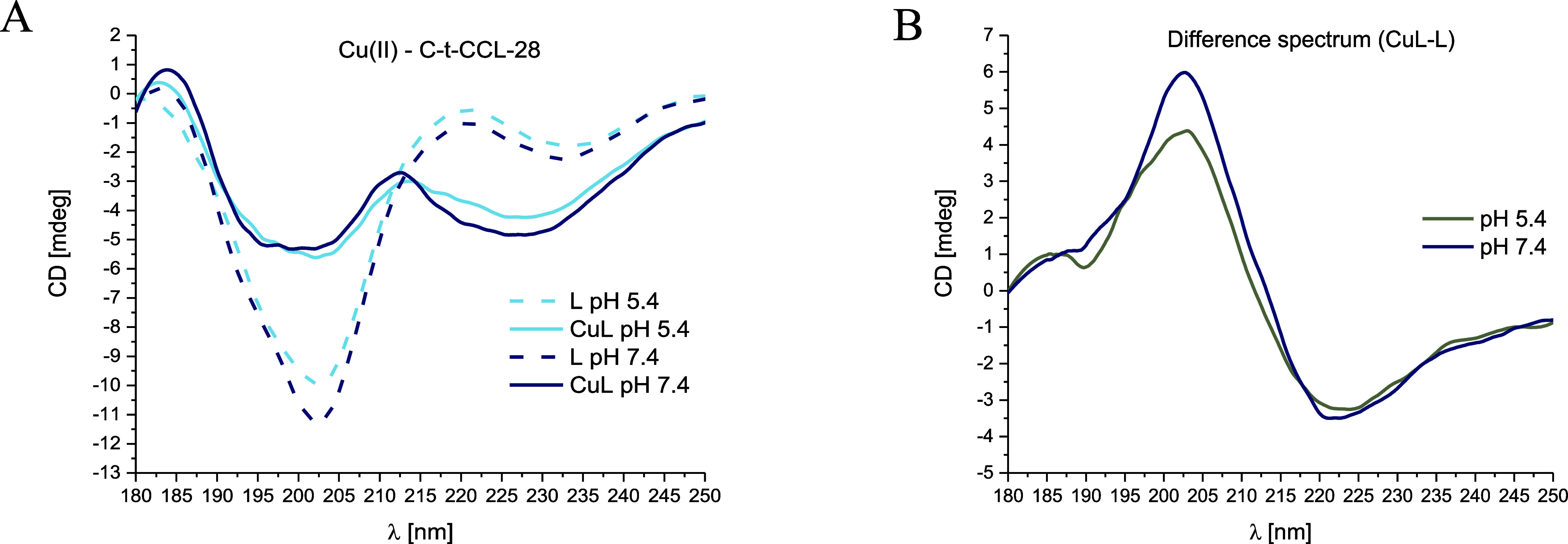
(A) Far-UV CD spectra at 180–250 nm at pH 5.4 and 7.4 for the Cu(II)–C-t-CCL-28 system in aqueous solution of 4 mM HClO_4_ with *I* = 0.1 M NaClO_4_; molar ratio M/L 0.9:1; the optical path length = 0.2 mm; C_L_ = 0.3 mM; the dashed lines correspond to the peptide spectra. B) Difference spectrum at pH 5.4 and 7.4.

Far-UV CD spectra of C-t-CCL-28 show random-coil conformation at the pH range of 3.4–11.4 (Figure S6). Cu(II) binding induces a significant change in the secondary structure, reducing the proportion of unstructured forms within the system, both at pH 5.4 and at pH 7.4 (Figures 8 and S7). The CD difference spectrum (which shows the subtracted spectrum of the peptide from the spectrum of the complex at the same pH value, Figure 8B) shows a tendency to form a β-sheet structure with characteristic bands at around 200 and 220 nm, which are the closest values to those found in the literature for β-sheet conformation.^43^ On the contrary, Zn(II) coordination did not trigger any structural changes, neither at pH 5.4 nor at pH 7.4 (Figures S8 and S9).

### Redox Activity

Cyclic voltammograms were recorded for the Cu(II)–C-t-CCL-28 complex in two potential ranges (Figure S10 A,B). Experiments were performed at pH 5.4 and 7.4, where [CuH_10_L]^8+^ and [CuH_8_L]^6+^ complex species predominated, respectively. Since at both of these pH values, 3N coordination mode with {3N_im_} donor set was predicted, the electrochemical behavior was similar. In the anodic potential range (Figure S10 A, from 0.4 to 0.9 V), irreversible tyrosine oxidation was generated by Tyr-22 and Tyr-28 present in the peptide sequence. Upon increasing the pH from 5.4 to 7.4, the oxidation signal was shifted from *E*_oxTyr_ ∼ 0.68 V to less positive value *E*_oxTyr_ ∼ 0.62 V, which is in good agreement with the literature data.^44,45^ In potentials ranging from 0.4 to −0.5 V (Figure S10A,B), the Cu(II)/(I) process was visible. At pH 5.4, a cathodic peak was observed at the potential *E*_red_ ∼ −0.19 V, associated with reduction of Cu(II) in complex, and an anodic peak at *E*_ox_ = +0.27 V related to the oxidation of Cu(I) in complex. The large anodic-to-cathodic peak difference (Δ*E* = 0.46 V) strongly suggests slow kinetics of the structural rearrangement in the tested complex. At pH 7.4, the Cu(II)/Cu(I) process occurred at the same potential value, while the electrooxidation signal was shifted to less positive value *E*_ox_ = +0.17 V. These results indicated that less energy is needed to oxidize Cu(I) in the [CuH_8_L]^6+^ complex than in the [CuH_10_L]^8+^ one. On the basis of the calculated formal potentials, *E*_f_ = (*E*_ox_ + *E*_red_)/2) *E*_f_ ∼ 0.04 V (pH 5.4) and *E*_f_ ∼ 0.01 V (pH 7.4), it can be concluded that the Cu(II)–C-t-CCL-28 complex may contribute to the ROS production.^46,47^

### ROS Detection

The ROS production was confirmed by a fluorescence test with CCA, which is a good detector for hydroxyl radicals generated chemically by the Cu(II)-mediated oxidation of ascorbic acid (Asc).^28^ The significant effect—the highest fluorescence intensity was observed in the obtained spectra at 452 nm (λ_em_ characteristic for 7-OH–CCA, an oxidation product resulting from HO^•^ trapping by CCA) after 5 min of measurement (Figure S11, C) and strongly suggests ROS generation by the Cu(II)–C-t-CCL-28 system under the studied conditions.

ROS generation was also confirmed through ascorbate consumption experiments using UV–vis spectroscopy. To evaluate the production of hydroxyl radicals (OH^•^) by Cu(II)–C-t-CCL-28 in the presence of ascorbate, three comparative systems were employed: (i) Cu(II) with ascorbate in buffer, (ii) Cu(II)–C-t-CCL-28 with ascorbate in buffer, and (iii) C-t-CCL-28 with ascorbate in buffer (Figure S12). The most pronounced oxidation of ascorbate occurred in the presence of free Cu(II), which aligns with the existing literature.^48^ However, Cu(II)–C-t-CCL-28 also demonstrated significant OH^•^ production in the presence of ascorbate, whereas the free peptide showed no substantial OH^•^ generation (Figure S12).

### Antimicrobial Activity

To evaluate the effectiveness of the C-t-CCL-28 peptide and its metal ion complexes against microbes, we used a broth microdilution test. This method helped to determine the MIC, indicating the lowest concentration at which the growth of tested microorganisms is inhibited. Considering saliva’s usual slightly acidic pH, ranging from 5.0 to 8.0, and varying among individuals’ health conditions,^49^ we examined the antimicrobial properties of the C-t-CCL-28 peptide and its metal ion complexes against six bacterial strains and one fungal strain at pH levels of 7.4 (Table 2) and 5.4 (Table 3).

**Table 2:** *In Vitro* Antibacterial and Anti-*Candida* Activity of Peptides/Complexes Determined as a MIC (μg/mL); Antimicrobial Assays Were Performed in 10 mM HEPES Buffer (pH 7.4)^a^

pH 7.4	*E. coli* ATCC 25922	*P. aeruginosa* ATCC 15422	*E. faecalis* ATCC 29212	*S. aureus* ATCC 25923	*S. mutans* PCM 2502	*S. sanguinis* PCM 2335	*C. albicans* SC5314
C-t-CCL-28	*n*/*d*	*n*/*d*	*n*/*d*	*n*/*d*	*n*/*d*	*n*/*d*	*n*/*d*
Cu(II)–C-t-CCL-28	*n*/*d*	*n*/*d*	*n*/*d*	*n*/*d*	*n*/*d*	*n*/*d*	*n*/*d*
Zn(II)–C-t-CCL-28	*n*/*d*	*n*/*d*	*n*/*d*	*n*/*d*	*n*/*d*	500	*n*/*d*
Chloramphenicol	1	500	4	15.625	62.5	62.5	
Fluconazole							1

a Chloramphenicol and fluconazole were used as a reference for the antibacterial and antifungal tests, respectively. Experiments were performed for peptide and its copper(II) and zinc(II) complexes. *n*/*d*, not determined within the concentration range used in this study.

**Table 3:** *In vitro* Antibacterial and Anti-*Candida* Activity of Peptides/Complexes Determined as a MIC (μg/mL); Antimicrobial Assays Were Performed in 10 mM MES Buffer (pH 5.4)^a^

pH 5.4	*E. coli* ATCC 25922	*P. aeruginosa* ATCC 15422	*E. faecalis* ATCC 29212	*S. aureus* ATCC 25923	*S. mutans* PCM 2502	*S. sanguinis* PCM 2335	*C. albicans* SC5314
C-t-CCL-28	*n*/*d*	*n*/*d*	500	500	250	500	31.25
Cu(II)–C-t-CCL-28	500	500	125	125	125	250	15.625
Zn(II)–C-t-CCL-28	500	500	125	125	62.5	125	15.625
Chloramphenicol	1	500	4	15.625	62.5	62.5	
Fluconazole							1

a Chloramphenicol and fluconazole were used as a reference for the antibacterial and antifungal tests, respectively. Experiments were performed for peptide and its copper(II) and zinc(II) complexes. *n*/*d*, not determined within the concentration range used in this study.

At pH 7.4, antimicrobial activity of C-t-CCL-28 is only observed against *S. sanguinis* after binding Zn(II), and the MIC value for this system is far too low for commercial use. However, at a lower pH (5.4), the effectiveness of C-t-CCL-28 in combating microbes is in all studied cases notably enhanced by the binding of metal ions (Table 3). Particular attention should be paid to the antifungal activity against *C. albicans*. For the peptide itself, it is already excellent, but after binding the metal ions, the antifungal activity becomes even more enhanced. The MIC values determined for the C-t-CCL-28’s complexes were 15.625 μg/mL for both Cu(II) and Zn(II) complexes. This study also compared the antibacterial properties of C-t-CCL-28 and its metal(II) complexes with those of a commonly used antibiotic, chloramphenicol, against the tested pathogens. The results indicate that the antibacterial effectiveness of the Zn(II)–C-t-CCL-28 complex is similar to that of chloramphenicol at comparable concentrations (Table 3). The overall increase in antimicrobial efficacy induced by Zn(II) in the investigated C-t-CCL-28 likely stems from alterations in the local charge of the complex. However, when it comes to the C-t-CCL-28 complex with Cu(II), the increase in antimicrobial activity may depend not only on the increase in the overall charge of the system but also on its structural change and redox activity. The produced ROS may oxidize the pathogen’s lipid bilayer, leading to its destruction.

**Figure 9:**
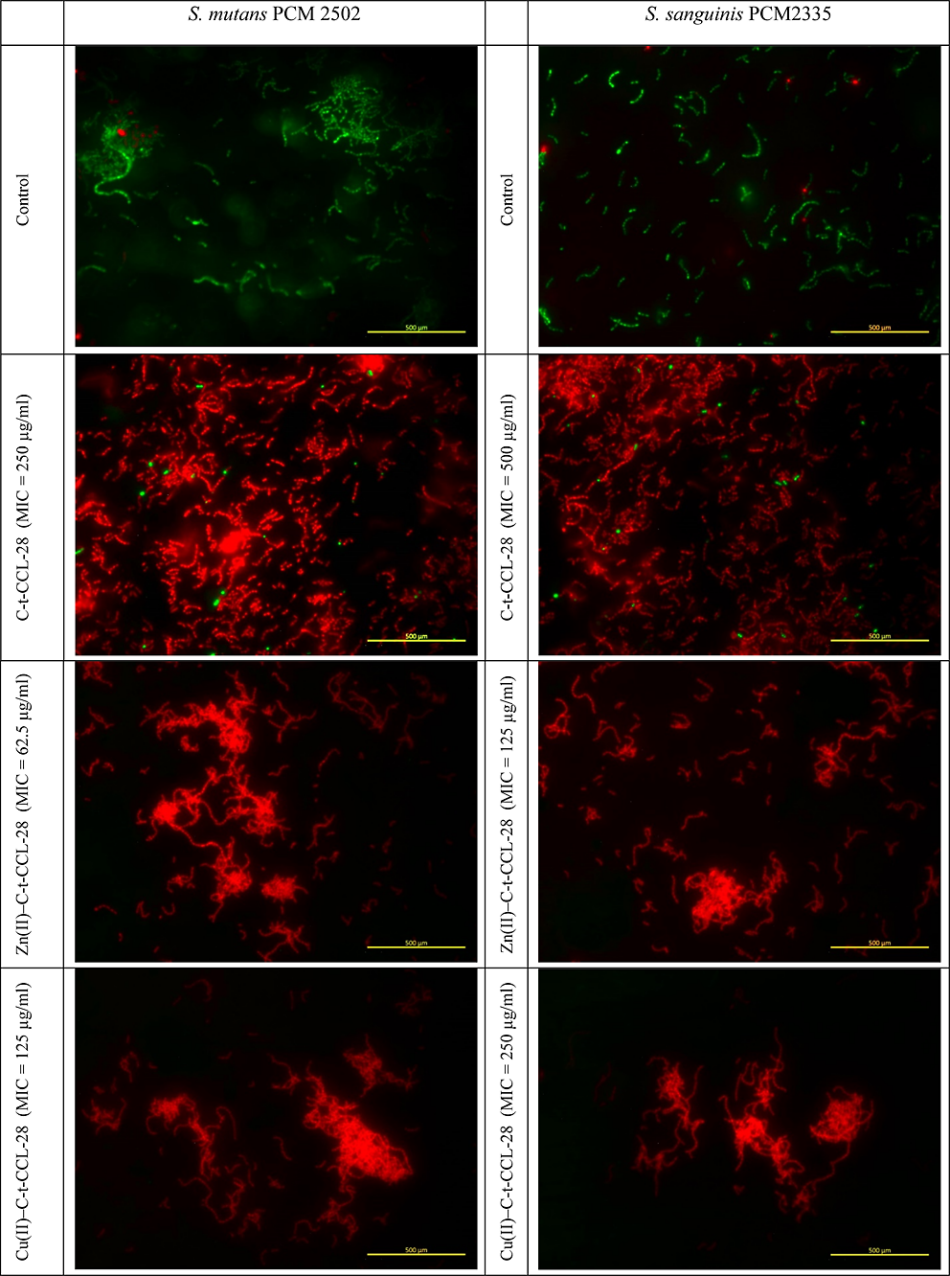
Fluorescence microscopy assays for the study of the viability of *S. mutans* and *S. sanguinis* treated with the C-t-CCL-28 fragment and its Cu(II) and Zn(II) complexes. Bacteria in green indicates live/healthy cells, whereas red is indicative of dead or membrane-damaged bacteria. Scale bar −500 μm.

Fluorescence images revealed the viability of bacteria after treatment with the C-t-CCL-28 fragment and its complexes against *S. mutans* and *S. sanguinis* at pH 5.4. The permeability of the cell membrane was visualized after staining the bacterial cells with two fluorescent nucleic acid stains, SYTO-9 and propidium iodide (PI). Live bacteria were stained with SYTO-9, emitting green fluorescence, while dead bacteria with compromised membranes were marked by PI, showing red fluorescence. In the control assays (Figure 9), mainly the green color is observed, which indicates a large number of live cells. The treatment with the C-t-CCL-28 fragment (Figure 9) led to an almost complete red color, which indicates dead cells. The Cu(II)–C-t-CCL-28 and Zn(II)–C-t-CCL-28 complexes were equally effective against *S. mutans* and *S. sanguinis*. Figure 9 shows the increase in PI staining (red fluorescence) due to the Cu(II)- and Zn(II)-complexes exposure. These results suggest that the C-t-CCL-28 fragment and its complexes effectively killed the bacterial cells. Destruction of bacterial cells, like *S. mutans* and *S. sanguinis*, is a key strategy for attacking inflammatory dental diseases, given the compromised efficacy of currently used antibiotics against antibiotic-resistant pathogens. Despite the evidence of cell membrane damage, this study does not exclude additional bactericidal mechanisms potentially exerted by the Cu(II)–C-t-CCL-28 and Zn(II)–C-t-CCL-28 complexes.

**Figure 10:**
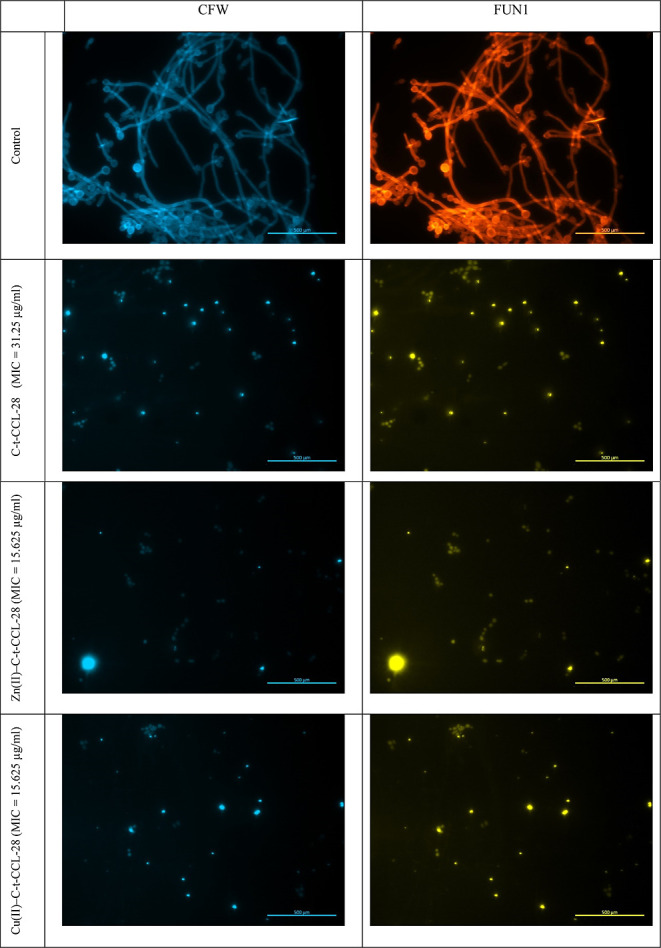
Viability of *C. albicans* SC5314 cells using CFW and FUN1. *C. albicans* viability was imaged via fluorescence microscopy. Scale bar, 500 μm.

To demonstrate the antifungal efficacy of the C-t-CCL-28 fragment and its complexes against *C. albicans* at pH 5.4, cellular visualization was conducted using fluorescence microscopy subsequent to staining with two fluorescent probes, calcofluor white (CFW) and FUN1. *Candida* cells were stained by CFW to identify the cell wall of yeast, which is composed of β-glucans. Concurrently with staining of *C. albicans* with CFW, green and red fluorescence of FUN1 was also applied. Areas fluorescing orange-red represent the presence of metabolically active microbial cells, whereas in dead cells, FUN1 remains in the cytosol and fluoresces yellow-green (Figure 10). As seen in Figure 10, exclusively viable *C. albicans* cells were observed, predominantly in filamentous forms linked to its pathogenesis. Upon introduction of C-t-CCL-28, a marked reduction in visible live cells was observed, accompanied by an increase in dead cells. Filamentous structures were no longer discernible, indicating significant deterioration of the target microorganisms. The addition of the Zn(II) and Cu(II) ions to the peptide causes a further reduction in the number of viable cells within the visual field, confirming the even better antifungal activity.

## Conclusions

It seems that nature is an expert in designing tailor-made AMPs, active exactly at pH that is often expected in the oral cavity (pH 5.4); the C-t-CCL-28 peptide is an excellent example. It is not pathogen-specific but rather effective against a wide range of bacteria and fungi, especially those commonly found in the dental plaque, such as *S. sanguinis* and *S. mutans*.

The relationship between coordination chemistry and the antimicrobial activity of Zn(II) and Cu(II) complexes with C-t-CCL-28 is a chemically fascinating phenomenon; in most cases, metal ions trigger or enhance the peptide’s activity. Notably, the best MIC values with clinical anti-*candidal* potency for both complexes were observed.

The Cu(II)–C-t-CCL-28 complex most likely has a redox-linked mode of action (it is able to generate ROS), while the explanation of the Zn(II)–C-t-CCL-28 mode of action remains not fully understood. Due to the substantial similarities in the amino acid sequence between C-t-CCL28 and Hst-5, as well as the binding of Zn(II) ions via highly similar motifs (HEXXXH in case of C-t-CCL-28 and HEXXH in Hst-5), it is suggested that they share a similar antimicrobial mechanism, most likely the one described by Campbell *et al*., in which the His-5′s antimicrobial activity is regulated by Zn(II) ion levels: (i) low Zn(II) enhances its activity against *C. albicans*, while (ii) high Zn(II) inhibits it, potentially allowing the host to modulate microbial balance during infection.^50^

Our findings are an important input into the understanding of the function of CCL-28, in the presence of Zn(II) and Cu(II) ions, and we do believe it may become a promising agent in the fight with candidiasis. This is significant to consider, given that oral candidiasis is a very common opportunistic infection of the oral mucosa.^51,52^ Fighting *S. mutans* is also crucial not only for oral health but also due to its implications in serious conditions such as heart valve inflammation, cerebral microbleeds, IgA nephropathy, and atherosclerosis.^53^ The complex with Zn(II) ions shows the same activity against *S. mutans* (at moderately acidic pH) as the commonly used chloramphenicol, which may constitute an excellent alternative to the use of this (quite toxic to humans^54,55^) antibiotic in the future.

## Supporting Information Available

Thermodynamic and spectroscopic data for proton and the Cu(II)–C-t-CCL-28; thermodynamic data for proton and the Zn(II)–C-t-CCL-28; mass spectra for the metal–peptide systems; EPR spectra for Cu(II)–C-t-CCL-28; far-UV CD spectra for C-t-CCL-28 and its metal complexes; CVs for Cu(II)–C-t-CCL-28 at 5.4 and 7.4; fluorescence curves of ROS production; and UV–vis spectra of ascorbate consumption (PDF)

## Abbreviations

AMP: antimicrobial peptides
Asc: ascorbic acid
ATCC: American Type Culture Collection
ATCUN: the amino terminal Cu(II)- and Ni(II)-binding motif
BHI: Brain Heart Infusion broth
CCA: coumarin-3-carboxylic acid
CCL-28: chemokine (C–C motif) ligand 28
CD: circular dichroism
C-t-CCL-28: C-terminal chemokine (C–C motif) ligand 28
CFW: calcofluor white
CV: cyclic voltammetry
EPR: electron paramagnetic resonance
MEC: mucosae-associated epithelial chemokine
MHB: Mueller–Hinton broth
MIC: minimal inhibitory concentration
OH: hydroxyl radical
PCM: polish collection of microorganisms
PI: propidium iodide
ROS: reactive oxygen species
YPD: Yeast Peptone Dextrose broth
7-OH–CCA: 7-hydroxycoumarin-3-carboxylic acid
