# Supplementary material for: Bioinorganic Chemistry Meets Microbiology: Copper(II) and Zinc(II) Complexes Doing the Cha-Cha with the C-t-CCL-28 Peptide, Dancing till the End of Microbes
Source: Inorg Chem. 2024 Oct 1;63(41):19105–16. doi: 10.1021/acs.inorgchem.4c02500 (PMC11483739; doi:10.1021/acs.inorgchem.4c02500)
Supplement: Supplementary file 1 — ic4c02500_si_001.pdf [file ic4c02500_si_001.pdf]

Supporting Information for:

Bioinorganic Chemistry Meets Microbiology: Copper(II) and Zinc(II) Complexes Doing the Cha-Cha with C-t-CCL-28 Peptide, dancing till the end of Microbes

Klaudia Szarszoń, [a] *Natalia Baran,* [b] *Paulina Śliwka,* [c] *Magdalena Wiloch,* [b] *Tomasz Janek,* [c] *Joanna Wątły* [a*]

[a] Faculty of Chemistry, University of Wrocław, F. Joliot-Curie 14, 50-383 Wrocław, Poland

[b] Institute of Physical Chemistry, Polish Academy of Sciences, Kasprzaka 44/52, 01-224 Warsaw, Poland

[c] Department of Biotechnology and Food Microbiology, Wrocław University of Environmental and Life Sciences, Chełmońskiego 37, 51-630, Wrocław, Poland

*joanna.watly2@uwr.edu.pl

Table of Contents:

Table S 1. Deprotonation constants and representative distribution diagram for C-t-CCL-28 peptide in aqueous solution of 4 mM $HClO_4$ with $I = 0.1$ M $NaClO_4$ dependent on pH values; $C_L = 0.4$ mM; T = 25 ˚C. [a]Constants are presented as cumulative log β_{jk} values. $\beta(H_jL_k) = [H_jL_k]/([H]^j[L]^k)$, in which [L] is the concentration of the fully deprotonated peptide. [b]pK_a values of the peptides were derived from cumulative constants: pK_a = log $\beta(H_jL_k)$ – log $\beta(H_{j-1}L_k)$. 3

Figure S 1. Mass spectra (ESI-MS) for the system Zn(II) – C-t-CCL-28 A) whole spectrum; example of B) experimental and C) simulated spectrum for $[ZnL]^{5+}$ complex with 1:1 stoichiometry....................4

Figure S 2. Mass spectra (ESI-MS) for the system Cu(II) – C-t-CCL-28 A) whole spectrum; example of B) experimental and C) simulated spectrum for $[CuL]^{5+}$ complex with 1:1 stoichiometry. 5

Figure S 3. Mass spectra (ESI-MS) for the system C-t-CCL-28 – Cu(II) – Zn(II) A) in the molar ratio 1:1:1; B) in the molar ratio 1:0.5:0.5.On the top whole spectra were presented and below them - the examples of experimental and simulated spectra for the region with 6+ charge. Additional signals in the spectra are sodium/potassium or sodium-potassium adducts for the ligand and complex forms............6

Figure S 4. pH-dependent EPR spectra for the Cu(II) – C-t-CCL-28 system in aqueous solution with the addition of ethylene glycol (30%) at temperature 77 K; [Cu(II)] = 0.001 M; molar ratio M:L equal to 0.9:1. 7

Table S 2. Thermodynamic and spectroscopic data for proton and the Cu(II) – C-t-CCL-28 in aqueous solution of 4 mM $HClO_4$ for each calculated complex species with the proposed coordination modes, C_L = 0.4 mM; molar ratio M:L – 0.9:1; I = 0.1 M $NaClO_4$; T = 25 ˚ C. 7

Figure S 5. Competition plot for Cu(II) and Zn(II) complexes with C-t-CCL-28 based on potentiometric data (Table 1), describing complex formation at different pH values in a hypothetical situation, in which equimolar amounts of the all reagents are mixed. Conditions: T = 25 ˚C , [Cu(II)]=[Zn(II)]=[C-t-CCL-28]=0.001 M. T = 25 ˚C 9

Figure S 6. Far-UV CD spectra at 180-250 nm for C-t-CCL-28 peptide in aqueous solution of 4 mM $HClO_4$ with $I = 0.1$ M $NaClO_4$; the optical path length = 0.2 mm; C_L = 0.3 mM. 10

Figure S 7. Far-UV CD spectra at 180-250 nm for Cu(II) – C-t-CCL-28 system in aqueous solution of 4 mM $HClO_4$ with I = 0.1 M $NaClO_4$; molar ratio M:L 0.9:1; the optical path length = 0.2 mm; C_L = 0.3 mM....................10

Figure S 8. Far-UV CD spectra at 180-250 nm for Zn(II) – C-t-CCL-28 system in aqueous solution of 4 mM $HClO_4$ with I = 0.1 M $NaClO_4$; molar ratio M:L 0.9:1; the optical path length = 0.2 mm; C_L = 0.3 mM....................11

Figure S 9. Far-UV CD spectra at 180-250 nm at pH 5.50 and 7.50 for Cu(II) – C-t-CCL-28 system in aqueous solution of 4 mM $HClO_4$ with I = 0.1 M $NaClO_4$; molar ratio M:L 0.9:1; the optical path length = 0.2 mm; C_L = 0.3 mM....................12

Figure S 10. CVs registered at pH 5.4 and 7.4 for Cu(II) – C-t-CCL-28 system in aqueous solution of 0.1 M KNO_3; molar ratio M:L 0.9:1; C_L = 0.3 mM; v = 20 mV/s....................12

Figure S 11. Fluorescence curves of ROS production. Fluorescence of the 7-OH-CCA (λ_{exc} = 395 nm, λ_{em} = 452 nm) as function of the time, showing the scavenging kinetics of HO· produced by Cu(II) (50 µM), ascorbate (200 µM) and peptide (60 µM) in phosphate-buffered solution (50mM, pH 5.4) with CCA (200 µM). The comparison of curves were presented: A) control sample with Cu(II), CCA and

Asc; control sample with peptide C-t-CCL-28, Cu(II) and CCA; sample with peptide C-t-CCL-28, Cu(II), CCA and Asc; B) control sample with Cu(II), CCA and Asc; sample with peptide C-t-CCL-28, Cu(II), CCA and Asc; C) control sample with peptide C-t-CCL-28, Cu(II) and CCA; sample with peptide C-t-CCL-28, Cu(II), CCA and Asc. The top row shows the measurement over the entire time range (1 hour), and the bottom row shows the measurement in the first 6 minutes of the experiment..13
Figure S 12. UV-Visible spectra showing ascorbate consumption by i) Cu(II) (black line); ii) Cu(II) – C-t-CCL-28 complex (red line) and iii) C-t-CCL-28 peptide (blue line) in phosphate buffer (pH 5.4) as a function of time. λ = 265 nm; [Asc] = 100 μM; [Cu(II)] = 8 μM; [C-t-CCL-28] =10 μM. 13

Table S 1. Deprotonation constants and representative distribution diagram for C-t-CCL-28 peptide in aqueous solution of 4 mM $HClO_4$ with I = 0.1 M $NaClO_4$ dependent on pH values; C_L = 0.4 mM; T = 25 ˚C. [a]Constants are presented as cumulative log β_{jk} values. $\beta(H_jL_k) = [H_jL_k]/([H]^j[L]^k)$, in which [L] is the concentration of the fully deprotonated peptide. [b]pK_a values of the peptides were derived from cumulative constants: $pK_a = \log \beta(H_jL_k) - \log \beta(H_{j-1}L_k)$.

species	log β_{jk} [a]	pK_a [b]	residue
$[H_{16}L]^{12+}$	125.51(1)	2.46	COOH
$[H_{15}L]^{11+}$	123.05(1)	3.75	Glu
$[H_{14}L]^{10+}$	119.3(1)	4.89	His
$[H_{13}L]^{9+}$	114.41(1)	5.59	His
$[H_{12}L]^{8+}$	108.82(1)	5.94	His
$[H_{11}L]^{7+}$	102.88(1)	6.33	His
$[H_{10}L]^{6+}$	96.55(1)	6.67	His
$[H_9L]^{5+}$	89.88(1)	7.21	His
$[H_8L]^{4+}$	82.67(1)	9.12	H_3N^+
$[H_7L]^{3+}$	73.55(1)	9.61	Tyr
$[H_6L]^{2+}$	63.94(1)	9.90	Tyr
$[H_5L]^{+}$	54.04(1)	10.28	Lys
$[H_4L]$	43.76(1)	10.49	Lys
$[H_3L]^{-}$	33.27(1)	10.90	Lys
$[H_2L]^{2-}$	22.37(1)		Lys

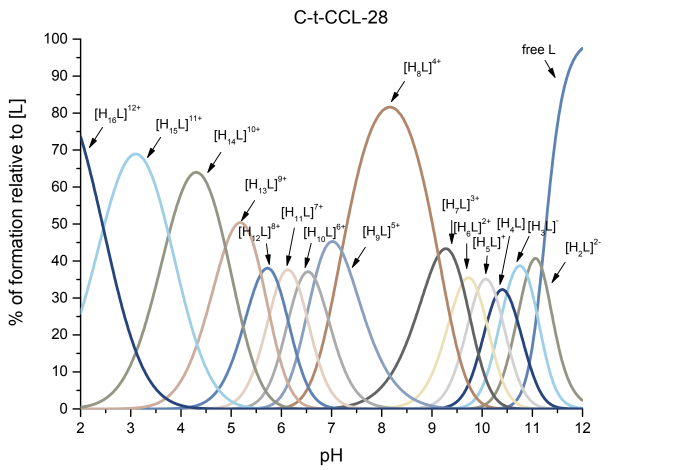

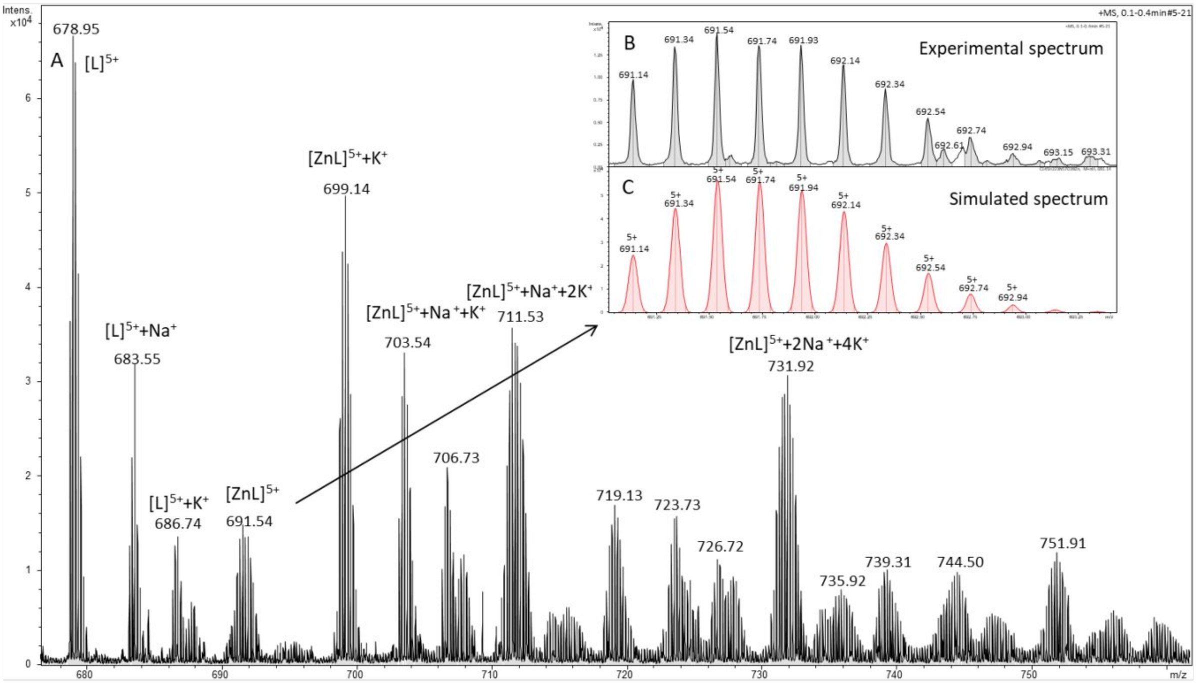

Figure S 1. Mass spectra (ESI-MS) for the system Zn(II) – C-t-CCL-28 A) whole spectrum; example of B) experimental and C) simulated spectrum for $[ZnL]^{5+}$ complex with 1:1 stoichiometry.

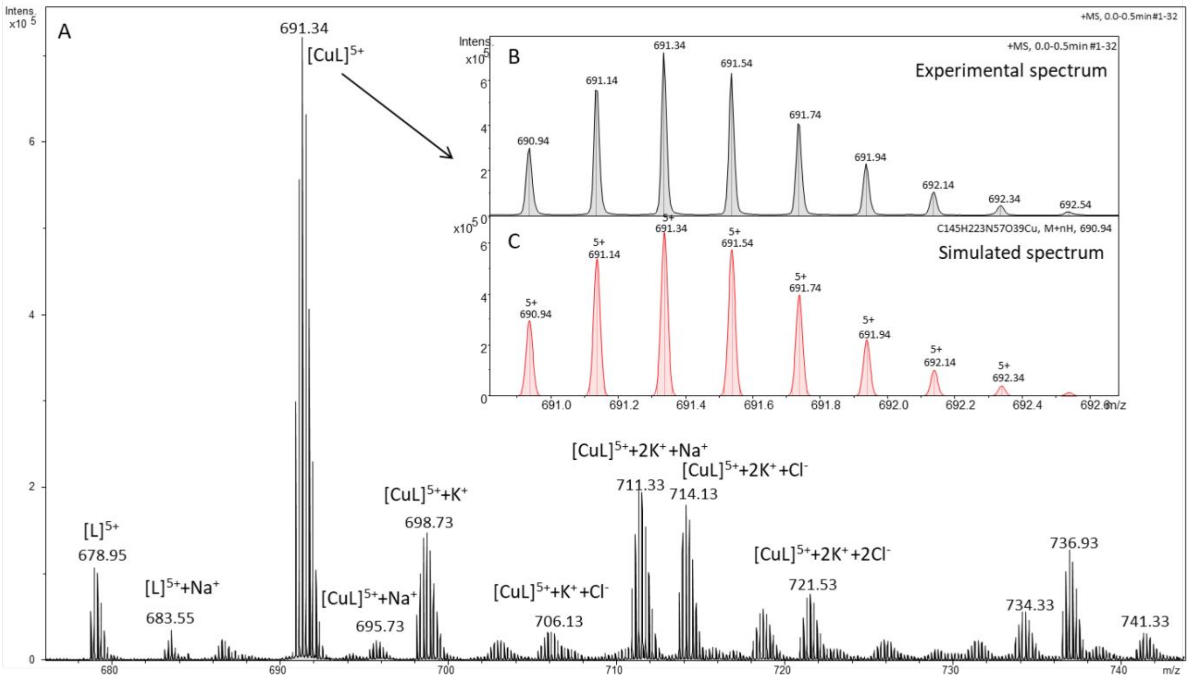

Figure S 2. Mass spectra (ESI-MS) for the system Cu(II) – C-t-CCL-28 A) whole spectrum; example of B) experimental and C) simulated spectrum for $[CuL]^{5+}$ complex with 1:1 stoichiometry.

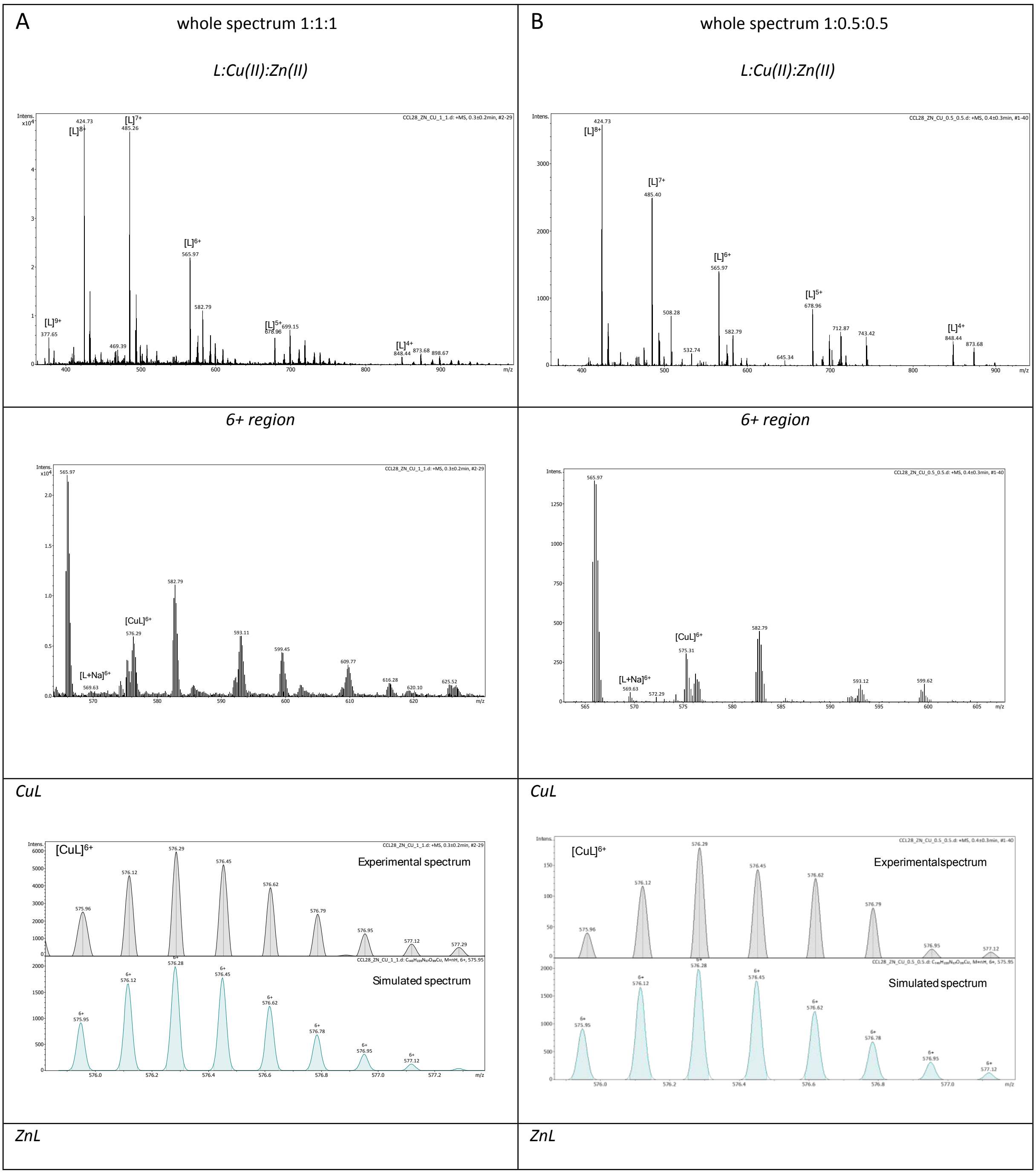

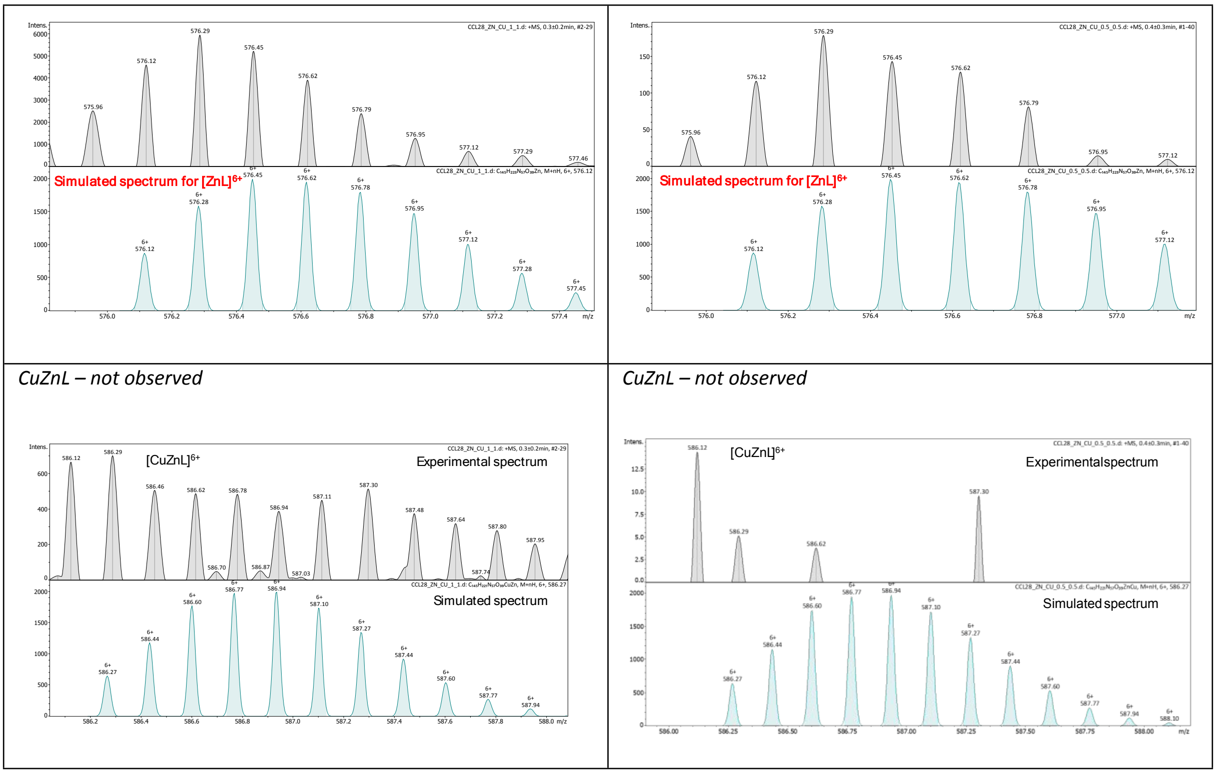

Figure S 3. Mass spectra (ESI-MS) for the system C-t-CCL-28 – Cu(II) – Zn(II) A) in the molar ratio 1:1:1; B) in the molar ratio 1:0.5:0.5.On the top whole spectra were presented and below them - the examples of experimental and simulated spectra for the region with 6+ charge. Additional signals in the spectra are sodium/potassium or sodium-potassium adducts for the ligand and complex forms.

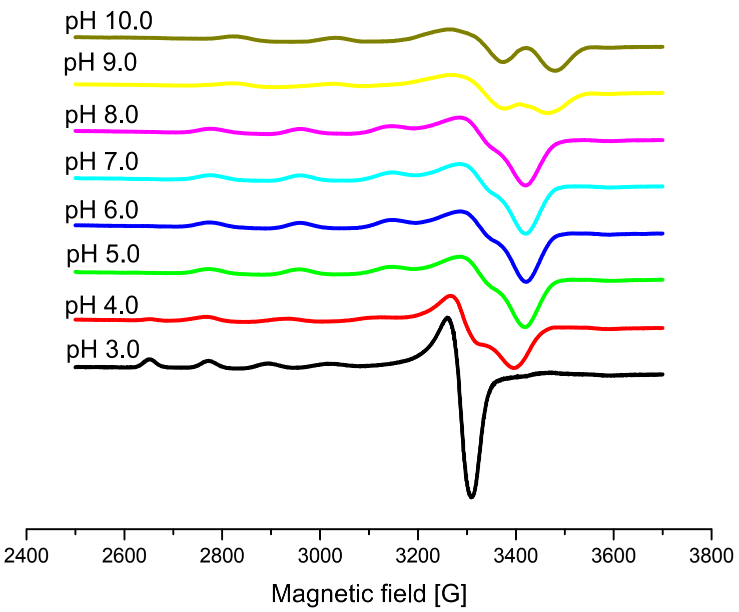

pH	A‖ (A_z) [G]	g‖ (g_z)	g⊥ (gx=gy)	MW Frequency
**3.0**	119	2.42	2.085	9.5778
**4.0**	173	2.26	2.080	9.5777
**5.0**	180	2.25	2.065	9.5777
**6.0**	181	2.24	2.060	9.577
**7.0**	182	2.24	2.060	9.5778
**8.0**	184	2.24	2.060	9.5772
**9.0**	200	2.19	2.050	9.5758
**10.0**	202	2.18	2.050	9.5753

Figure S 4. pH-dependent EPR spectra for the Cu(II) – C-t-CCL-28 system in aqueous solution with the addition of ethylene glycol (30%) at temperature 77 K; [Cu(II)] = 0.001 M; molar ratio M:L equal to 0.9:1.

Table S 2. Thermodynamic and spectroscopic data for proton and the Cu(II) – C-t-CCL-28 in aqueous solution of 4 mM $HClO_4$ for each calculated complex species with the proposed coordination modes, C_L = 0.4 mM; molar ratio M:L – 0.9:1; I = 0.1 M $NaClO_4$; T = 25 ˚ C.

Complex species	Complex species			UV-Vis		CD		EPR		Coordination mode
	logβ	pK_a	pH	nm	ε[$M^{-1}cm^{-1}$]	nm	Δ ε [$M^{-1}cm^{-1}$]	A‖ [G]	g‖	

**$[CuH_{12}L]^{10+}$**	115.32(1)		4.06	644	30.18	240	-3.06	173	2.26	2N<br>{$2N_{im}$}
**$[CuH_{11}L]^{9+}$**	110.84(2)	4.48	4.69	628	40.43	241	-3.15			2N<br>{$2N_{im}$}
**$[CuH_{10}L]^{8+}$**	106.15(2)	4.69	5.29	598	58.07	242	-3.35	180	2.25	3N<br>{$3N_{im}$}
**$[CuH_{9}L]^{7+}$**	100.31(2)	5.84	6.08	596	58.31	242	-3.43	181	2.24	3N<br>{$3N_{im}$}
**$[CuH_{8}L]^{6+}$**	93.96(2)	6.35	7.51	590	62.89	243	-3.13	182	2.24	3N<br>{$3N_{im}$}
**$[CuH_{6}L]^{4+}$**	77.47(2)		8.55	542	82.97	241<br>264<br>289<br>325	-1.39<br>0.05<br>-0.29<br>0.27	184	2.24	3N<br>{$3N_{im}$}
**$[CuH_{4}L]^{2+}$**	59.9(2)		9.28	522	100.23	238<br>258<br>287<br>320<br>524<br>703	-0.26<br>0.62<br>-0.38<br>0.52<br>-0.31<br>0.02	200	2.19	4N<br>{$3N_{im} + N_{am}$}
**$[CuH_{2}L]$**	40.47(3)		10.05	516	107.02	260<br>281<br>316<br>524<br>702	0.40<br>-0.30<br>0.41<br>-0.37<br>0.02	202	2.18	4N<br>{$N_{im} + 3N_{am}$}
**$[CuL]^{2-}$**	19.84(2)		10.69	516	111.14	260<br>278<br>311<br>519<br>701	0.25<br>-0.38<br>0.39<br>-0.39<br>0.03			4N<br>{$N_{im} + 3N_{am}$}
**$[CuH_{-2}L]^{4-}$**	-2.21(4)		11.16	516	114.07	263<br>280<br>311<br>519<br>701	0.23<br>-0.28<br>0.39<br>-0.40<br>0.03			4N<br>{$N_{im} + 3N_{am}$}
**$[CuH_{-3}L]^{5-}$**	-13.38(4)	11.17								4N<br>{$N_{im} + 3N_{am}$}

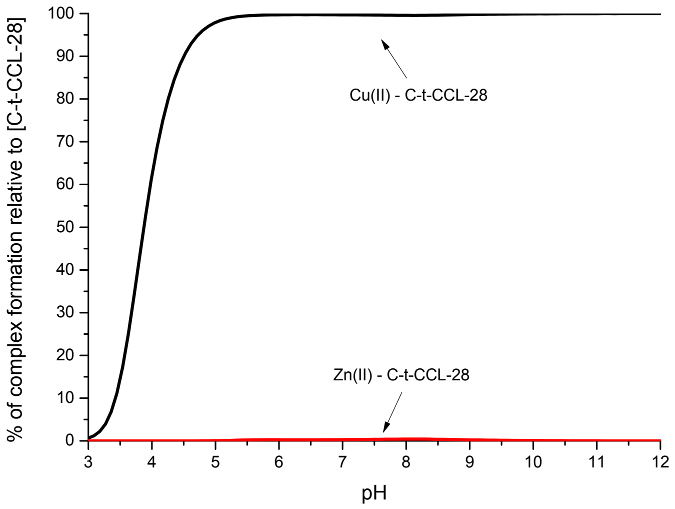

Figure S 5. Competition plot for Cu(II) and Zn(II) complexes with C-t-CCL-28 based on potentiometric data (Table 1), describing complex formation at different pH values in a hypothetical situation, in which equimolar amounts of the all reagents are mixed. Conditions: T = 25 ˚C , [Cu(II)]=[Zn(II)]=[C-t-CCL-28]=0.001 M. T = 25 ˚C

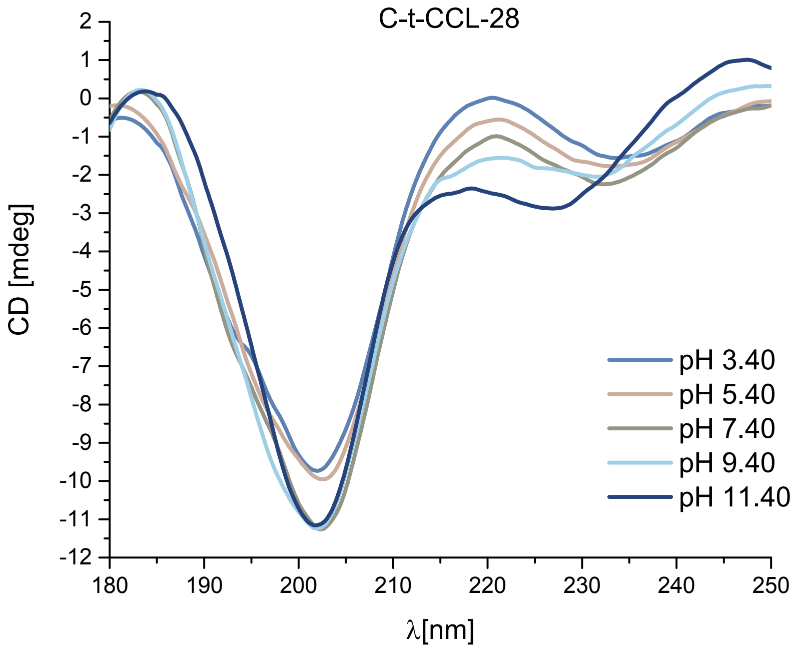

Figure S 6. Far-UV CD spectra at 180-250 nm for C-t-CCL-28 peptide in aqueous solution of 4 mM $HClO_4$ with I = 0.1 M $NaClO_4$; the optical path length = 0.2 mm; C_L = 0.3 mM.

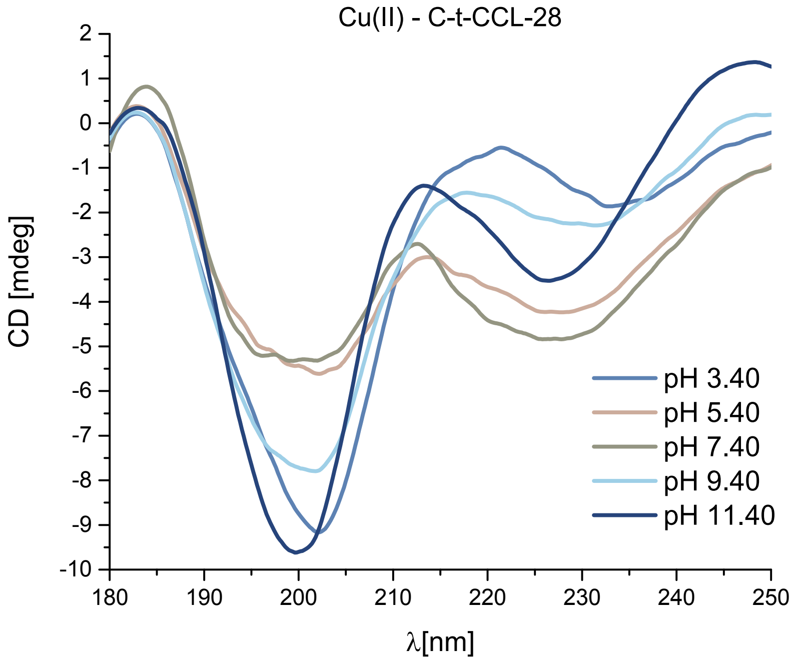

Figure S 7. Far-UV CD spectra at 180-250 nm for Cu(II) – C-t-CCL-28 system in aqueous solution of 4 mM $HClO_4$ with I = 0.1 M $NaClO_4$; molar ratio M:L 0.9:1; the optical path length = 0.2 mm; C_L = 0.3 mM.

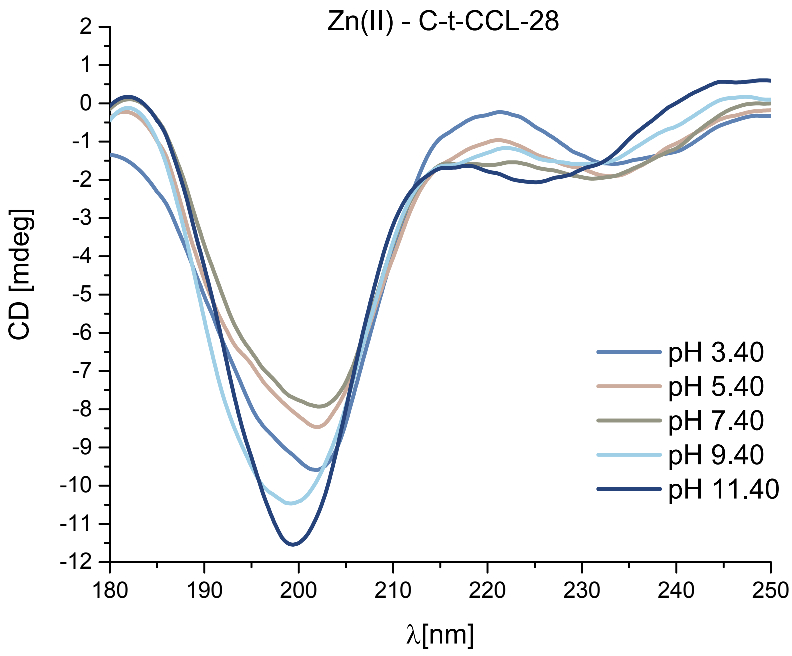

Figure S 8. Far-UV CD spectra at 180-250 nm for Zn(II) – C-t-CCL-28 system in aqueous solution of 4 mM $HClO_4$ with $I = 0.1$ M $NaClO_4$; molar ratio M:L 0.9:1; the optical path length = 0.2 mm; $C_L = 0.3$ mM.

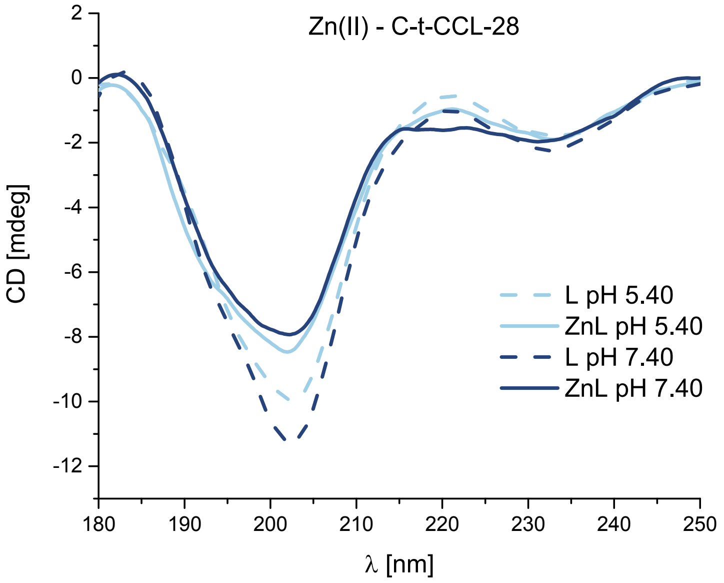

Figure S 9. Far-UV CD spectra at 180-250 nm at pH 5.50 and 7.50 for Cu(II) – C-t-CCL-28 system in aqueous solution of 4 mM $HClO_4$ with I = 0.1 M $NaClO_4$; molar ratio M:L 0.9:1; the optical path length = 0.2 mm; C_L = 0.3 mM.

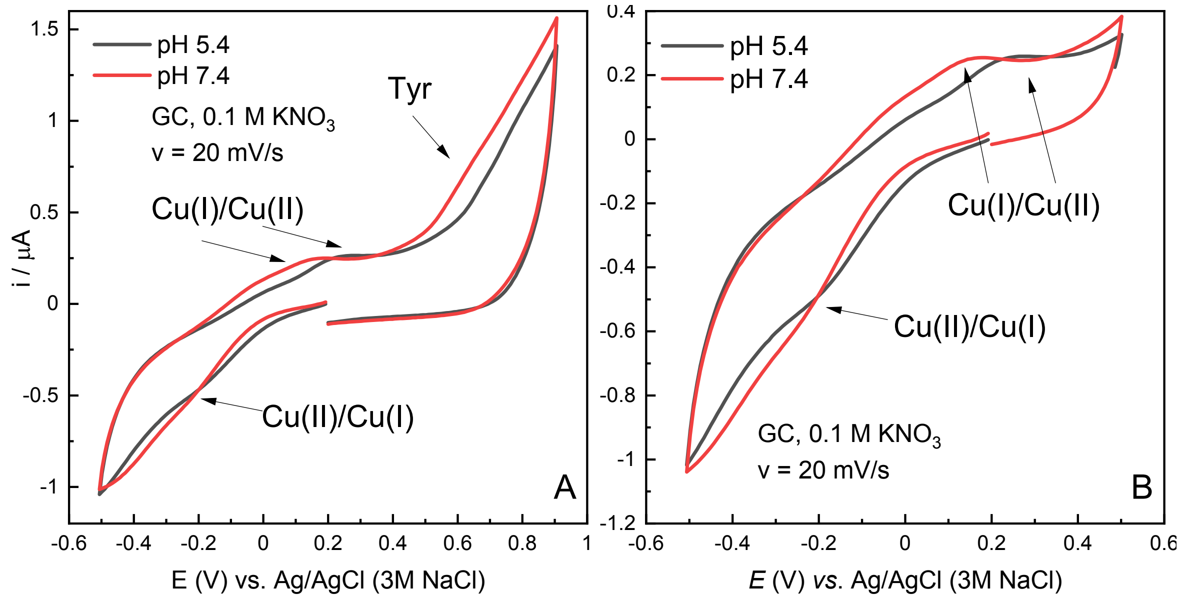

Figure S 10. CVs registered at pH 5.4 and 7.4 for Cu(II) – C-t-CCL-28 system in aqueous solution of 0.1 M KNO_3; molar ratio M:L 0.9:1; C_L = 0.3 mM; v = 20 mV/s.

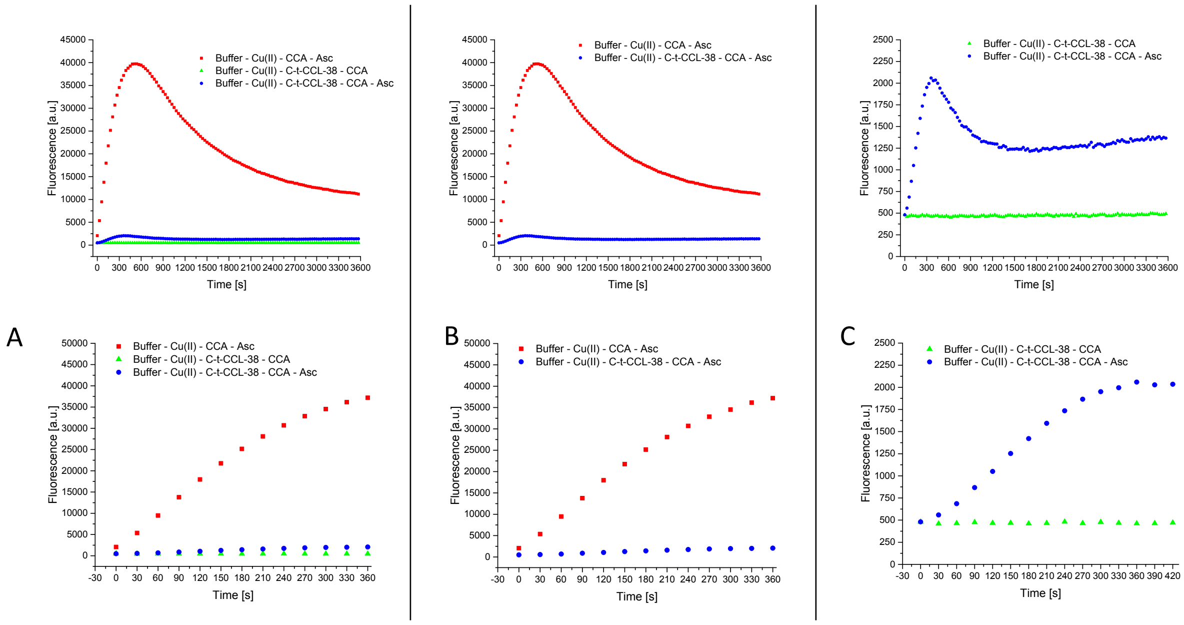

Figure S 11. Fluorescence curves of ROS production. Fluorescence of the 7-OH-CCA (λ_{exc} = 395 nm, λ_{em} = 452 nm) as function of the time, showing the scavenging kinetics of HO· produced by Cu(II) (50 μM), ascorbate (200 μM) and peptide (60 μM) in phosphate-buffered solution (50mM, pH 5.4) with CCA (200 μM). The comparison of curves were presented: A) control sample with Cu(II), CCA and Asc; control sample with peptide C-t-CCL-28, Cu(II) and CCA; sample with peptide C-t-CCL-28, Cu(II), CCA and Asc; B) control sample with Cu(II), CCA and Asc; sample with peptide C-t-CCL-28, Cu(II), CCA and Asc; C) control sample with peptide C-t-CCL-28, Cu(II) and CCA; sample with peptide C-t-CCL-28, Cu(II), CCA and Asc. The top row shows the measurement over the entire time range (1 hour), and the bottom row shows the measurement in the first 6 minutes of the experiment.

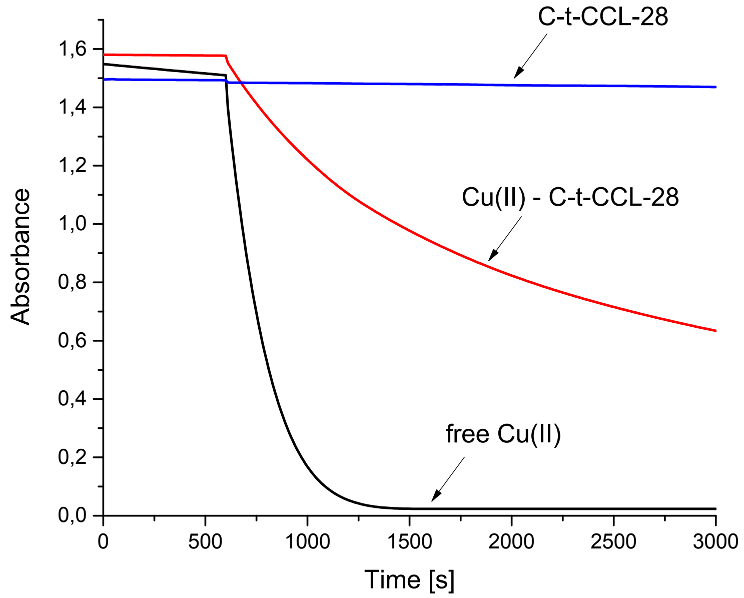

Figure S 12. UV-Visible spectra showing ascorbate consumption by i) Cu(II) (black line); ii) Cu(II) – C-t-CCL-28 complex (red line) and iii) C-t-CCL-28 peptide (blue line) in phosphate buffer (pH 5.4) as a function of time. λ = 265 nm; [Asc] = 100 μM; [Cu(II)] = 8 μM; [C-t-CCL-28] =10 μM.
